# Thermal mismatches explain consumer–resource dynamics in response to environmental warming

**DOI:** 10.1002/ece3.10179

**Published:** 2023-06-13

**Authors:** Soraya Álvarez‐Codesal, Cara A. Faillace, Alexandre Garreau, Elvire Bestion, Alexis D. Synodinos, José M. Montoya

**Affiliations:** ^1^ Theoretical and Experimental Ecology Station CNRS Moulis France; ^2^ Present address: Department of Biological Sciences University of Pittsburgh Pittsburgh Pennsylvania USA

**Keywords:** climate warming, consumer–resource interactions, interaction strength, phytoplankton physiology, temperature dependence, thermal performance curves, zooplankton physiology

## Abstract

Changing temperatures will impact food webs in ways we yet to fully understand. The thermal sensitivities of various physiological and ecological processes differ across organisms and study systems, hindering the generation of accurate predictions. One step towards improving this picture is to acquire a mechanistic understanding of how temperature change impacts trophic interactions before we can scale these insights up to food webs and ecosystems. Here, we implement a mechanistic approach centered on the thermal sensitivity of energetic balances in pairwise consumer–resource interactions, measuring the thermal dependence of energetic gain and loss for two resource and one consumer freshwater species. Quantifying the balance between energy gain and loss, we determined the temperature ranges where the balance decreased for each species in isolation (intraspecific thermal mismatch) and where a mismatch in the balance between consumer and resource species emerged (interspecific thermal mismatch). The latter reveals the temperatures for which consumer and resource energetic balances respond either differently or in the same way, which in turn informs us of the strength of top‐down control. We found that warming improved the energetic balance for both resources, but reduces it for the consumer, due to the stronger thermal sensitivity of respiration compared to ingestion. The interspecific thermal mismatch yielded different patterns between the two consumer–resource pairs. In one case, the consumer–resource energetic balance became weaker throughout the temperature gradient, and in the other case it produced a U‐shaped response. By also measuring interaction strength for these interaction pairs, we demonstrated the correspondence of interspecific thermal mismatches and interaction strength. Our approach accounts for the energetic traits of both consumer and resource species, which combined produce a good indication of the thermal sensitivity of interaction strength. Thus, this novel approach links thermal ecology with parameters typically explored in food‐web studies.

## Graphical abstract

Changing temperatures will impact food webs in ways we are yet to fully understand, as different physiological and ecological processes show different sensitivities to changing temperatures. After evaluating the thermal sensitivities of energy gain and loss parameters for consumer and resource species we found that when the energetic balances (i.e., the energy gain‐to‐loss ratio) of both interacting species declined with temperature, interaction strength increased with temperature, but interaction strength instead decreased, when the resources' energetic balance increased while that of the consumer declined.

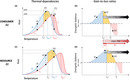

## 1 INTRODUCTION

Climate change is expected to increase mean surface temperatures between 1.5 and 5°C over the next 80 years (IPCC, 2021). This presents an important challenge across levels of biological organization as temperature regulates important ecological processes. Temperature affects growth, reproduction, development, and survival rates at the population level (Adamczuk, 2020; Dell et al., 2011; Frances et al., 2017), alters community size structure by benefiting smaller taxa (Daufresne et al., 2009; Yvon‐Durocher et al., 2011) and impacts several functions such as decomposition, productivity, and respiration at the ecosystem level (Yvon‐Durocher et al., 2010). Yet, we are far from being able to generate precise predictions on the effects of environmental warming on species and communities (Synodinos et al., 2021).

**FIGURE 1:**
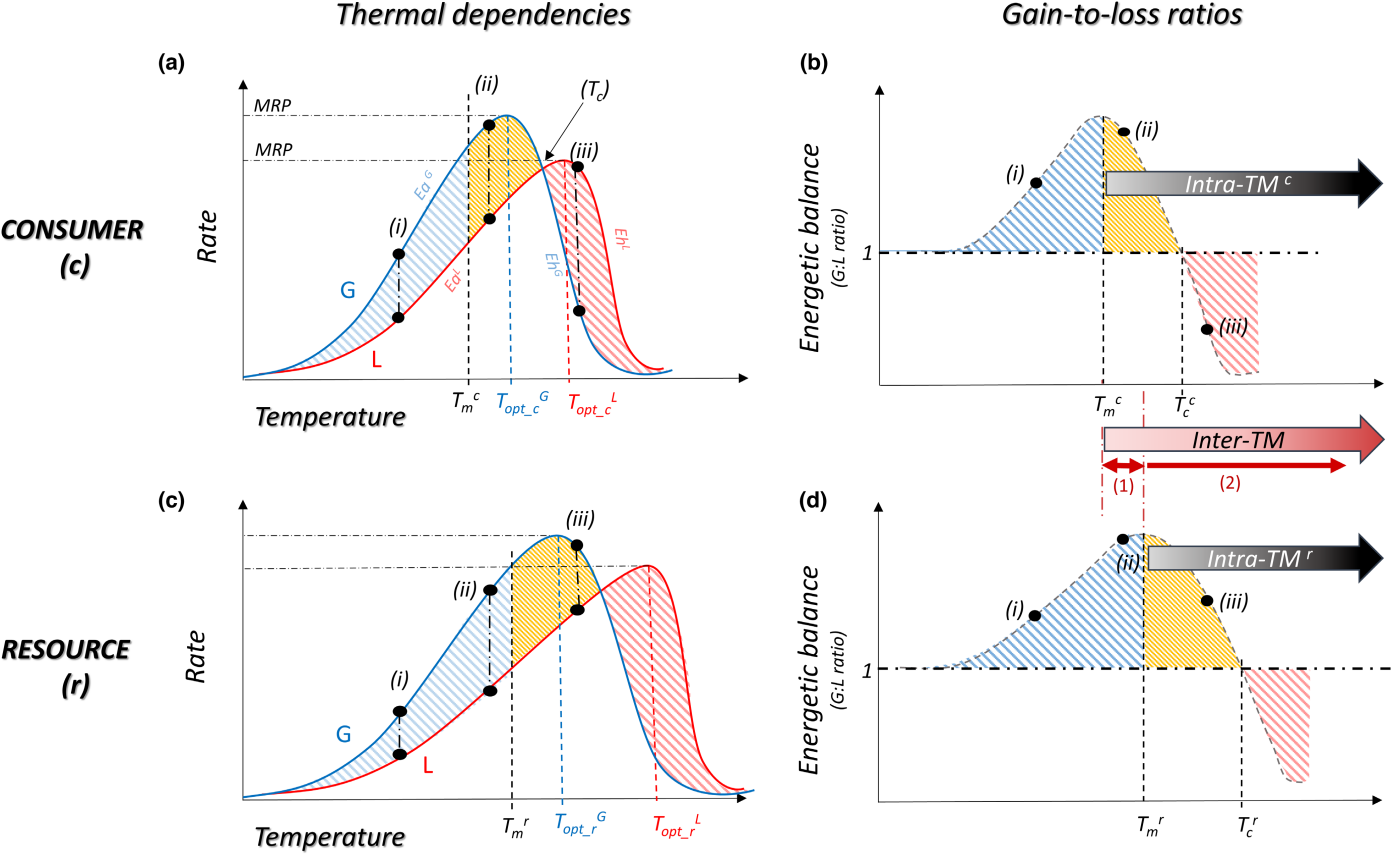
Species thermal mismatches of interacting consumer (a and b) and resource (c and d) species. (a) and (c) represent Thermal Performance Curves (TPCs) of energy gain (*G*, in blue) and loss (*L*, in red) parameters. The slope of the rate is the activation energy (*E*_a_), the temperature at which the rate peaks is the optimum temperature (*T*_opt_), the slope of the rate after the peak is the deactivation energy (*E*_h_), the maximal value of rate performance occurs at *MRP*, and the temperature of energy gain rate inflection ($T_{m}^{i}$, of consumer, *c*, or resource, *r*) is the maximum value of the gain‐to‐loss ratio. (b) and (d) represent the *G*/*L* ratio calculated from thermal dependencies of energy gain and loss depicted in (a) and (c). Blue‐shaded areas correspond to a positive energetic balance where energy gain increases faster with temperature than energy loss. Orange‐shaded areas correspond to a decreasing but still positive energetic balance with increasing temperatures. Red‐shaded areas correspond to a negative energetic balance, that is, where energy loss exceeds energy gain. Vertical dashed lines depict the temperature at which the G/L ratio is maximized (*T*_m_) and the crossover temperature (*T*_c_) where energy gain equals energy loss (ratio = 1). At low temperatures (i), for both species, the energetic balance of the organism increases with increasing temperature and gain exceeds loss. At intermediate temperatures (ii), energy gain exceeds loss for both species, but the ratio declines for the consumer, leading to a decline in the energy balance and the emergence of intraspecific thermal mismatches (Intra‐TM). At high temperatures (iii), energy loss for the consumer exceeds energy gain and the ratio falls below one, so the organism cannot fulfill its energetic demands. The resource also suffers from energetic deficits, but the G/L ratio still remains over one. Interspecific thermal mismatches (Inter‐TM) arise at temperatures at which one of the two interacting species starts to suffer from energetic deficits while the other does not. We distinguish two distinct thermal regions within the Inter‐TM: (1) where energetic balances differ between interacting species, and (2) where the energetic balances of both species decline with increasing temperature.

How organisms respond to environmental warming represents a complex question because temperature affects multiple biological rates simultaneously. Moreover, these rates can respond differently to temperature. To aggregate this information, one can measure how temperature alters an organism's energy gain and loss (Yodzis & Innes, 1992). In general, energy loss (e.g., respiration rate, metabolism, exudation, excretion) increases with temperature following either a monotonic (Brown et al., 2004; Goss & Bunting, 1980; Iles, 2014; Lemoine & Burkepile, 2012; Rall et al., 2010; Schulte et al., 2011; Vucic‐Pestic et al., 2011) or unimodal trend (Alcaraz et al., 2014; Booth et al., 2021; Padfield et al., 2016; Schaum et al., 2018; Schulte, 2015). Energy gain (e.g., nutrient acquisition, ingestion rate, attack rate) can similarly increase monotonically (Archer et al., 2019; Binzer et al., 2016; Fussmann et al., 2014; Rall et al., 2010; Vucic‐Pestic et al., 2011) or follow a unimodal trend (Alcaraz et al., 2014; Betini et al., 2019; Englund et al., 2011; Lemoine & Burkepile, 2012; Sentis et al., 2012; Uszko et al., 2017; West & Post, 2016). However, energy gain can also be invariant to temperature (Iles, 2014; Rall et al., 2012; Vucic‐Pestic et al., 2011). The ratio between energy gain and loss (i.e., G/L ratio in Figure 1b,d) can explain how species' energetic balance changes across temperatures, which ultimately affects individual fitness and population dynamics (Gilbert et al., 2014; Rall et al., 2010; Vucic‐Pestic et al., 2011).

Thermal mismatches and asymmetries of species are becoming the focus of recent research (Boukal et al., 2019; Dell et al., 2014; Gibert et al., 2022). Based on the change of energy gain and loss with increasing temperature we define intraspecific thermal mismatches to describe a negative effect on species’ energetic balance due to warming (intra‐TMs in Figure 1). Such mismatches may reflect two aspects of an energetic imbalance. First, when the G/L ratio decreases, the energetic balance is reduced, with consequences on the organism's maintenance, growth, and reproduction. The second aspect concerns the case where energy losses exceed gains, that is, when the G/L ratio is less than one. This can indicate an acute energetic limitation, which in the long term will lead to extinction. The characterization of intraspecific thermal mismatches can be the first step toward predicting responses to the warming of interacting species.

While intraspecific thermal mismatches are well‐characterized for consumers (Alcaraz et al., 2014; Iles, 2014; Lemoine & Burkepile, 2012; Rall et al., 2010; Vucic‐Pestic et al., 2011), they remain relatively understudied for resource species (Barton et al., 2020; Padfield et al., 2016). Moreover, empirical research on energetic balances of consumer–resource pairs remains scarce (Mertens et al., 2015). It is unclear whether intraspecific thermal mismatches of interacting species show similar trends with warming. If consumers and resources exhibit similar intraspecific patterns, then one can infer that the energetic balance of their interaction should remain unchanged with increasing temperatures. If, however, the energetic balances—and intraspecific thermal mismatches—of interacting consumer and resource species differ as temperature increases, interspecific thermal mismatches materialize (Inter‐TMs in Figure 1). Here, two distinct thermal regions can be identified within the Inter‐TM: (1) where energetic balances differ between interacting species and (2) where the energetic balances of both species decline with increasing temperature. Our definition of mismatch is hence broad, and it encapsulates thermal regions where both species have either different responses or different rates of response. Thus, the characterization of the thermal dependencies of both interacting species’ energetic balances can reveal potential interspecific thermal mismatches. These will provide information on how temperature change impacts the structure (e.g., biomass distribution) and dynamics of food webs through changes in energetic fluxes and interaction strength (Gilbert et al., 2014; Rip & Mccann, 2011).

Interaction strength is an important metric for predicting the temporal dynamics of resource and consumer populations, by quantifying the impact of the consumer on the resource's density (Berlow et al., 2009; Wootton & Emmerson, 2005). Increased interaction strength tends to correlate with lower stability, as the higher top‐down pressure makes dynamics more prone to fluctuations (McCann et al., 1998; Nilsson et al., 2018; Rip & Mccann, 2011) which could be linked to stochastic extinctions. However, the temperature dependence of interaction strength shows contrasting, sometimes contradictory results. Different studies have shown the thermal dependence of interaction strength to be monotonically increasing, monotonically decreasing, or hump‐shaped (Amarasekare, 2015; Fussmann et al., 2014; Gilbert et al., 2014; Rall et al., 2010; Synodinos et al., 2021; Vucic‐Pestic et al., 2011).

We hypothesize that the effects of warming on interaction strength can be predicted from interspecific thermal mismatches. In particular, the ratio between the energetic balances of the consumer and the resource (i.e., the Consumer–Resource Energetic Balance, CREB) provides a good proxy of interaction strength. The logic can be illustrated with an example (Box 1). In a situation where increasing temperature causes larger energetic constraints for the consumer than for the resource, the consumer's effect on resource densities should weaken as temperatures increase. More specifically, in this situation, the consumer energetic balance should decline first at lower temperatures relative to the energetic balance of the resource, which should lead to a declining pattern for their CREB (in logarithmic form) with warming. Thus, we hypothesize that the effects of temperature on the energetic constraints of the consumer relative to on the resource explains how temperature affects the impact of consumers on resources.

**BOX 1:**
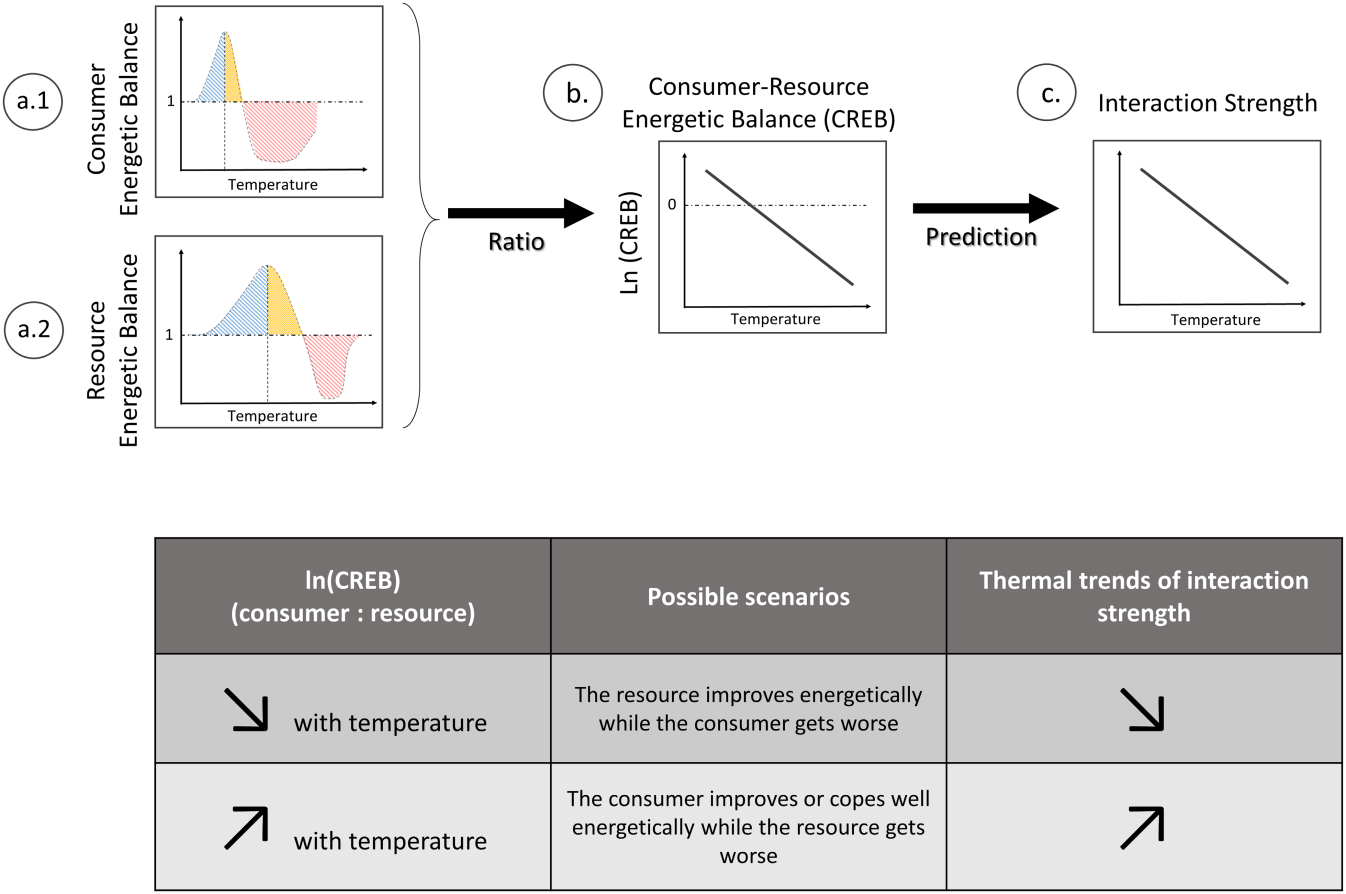
Hypothesis linking thermal responses of interacting species' thermal traits and trends of Interaction strength with temperature. We hypothesize that the response to temperature of Interaction Strength (IS) (i.e., per capita effect of the consumer on the resource densities) can be predicted by the differential responses of the energetic traits of consumers and resources. To do so, we propose to calculate the species Energetic Balances (EB), as the ratio of energy gain to energy loss for both the resource (a.1) and the consumer (a.2). From these, we calculate the natural logarithm of the Consumer–Resource Energetic Balance (CREB, b) for the interacting pair dividing the energetic balance of the consumer (a.1) by the energetic balance of the resource (a.2). Then, we hypothesize that the trends of the CREB predict the trends of interaction strength with temperature (c). We provide two possible scenarios as examples in the table above. The first scenario corresponds to panels (a.1, a.2, and b), where the CREB declines with increasing temperature because the resource improves energetically with temperature, while the consumer experiences energetic restrictions. Consequently, we predict that the consumer will exert less effect on the resource population as temperature increases, and hence interaction strength, measured as the log response ratio (ln[resource densities in the absence of consumers/resource densities in the presence of consumers]) will decrease with temperature. The second scenario represents a situation where the CREB increases with warming because the consumer improves energetically with temperature, while the resource experiences energetic restrictions. Here, we predict that consumers will exert a stronger effect on the resource biomass' densities as temperature increases, and hence interaction strength will increase with temperature.

We aim to answer three specific questions. First, does environmental warming cause intraspecific thermal mismatches in consumers and resources as a result of differences in thermal dependencies of energy gain and loss? Second, in consumer–resource interactions can high temperatures yield interspecific mismatches? And third, can interspecific thermal mismatches and consumer–resource energetic balances predict and provide a mechanistic understanding of the thermal dependence of interaction strength?

## 2 MATERIALS AND METHODS

### 2.1 Study system and experimental design

We focused on one consumer, *Daphnia pulex*, and two algal resource species, *Chlamydomonas reinhardtii* and *Desmodesmus* sp., isolated from shallow freshwater open‐air experimental ponds in Dorset, UK, between January and October 2019. These taxa are cosmopolitan and keystone freshwater species. After isolation, we maintained the organisms in semi‐continuous batch cultures in freshwater medium in incubators until experiments (see Methods S.1 in Data S1).

We measured thermal dependencies of physiological rates related to energy gain (i.e., ingestion, net photosynthesis) and loss (i.e., respiration) for the algae species and for *Daphnia* feeding on each resource separately in basal conditions at eight temperatures. For the algae, temperatures were 14, 18, 22, 26, 30, 34, 38, and 42°C, while temperatures were 14, 18, 22, 26, 30, 32, 34, and 36°C for *Daphnia*. The disparity in tested temperatures was linked to differences in thermal traits of the species found in previous pilot experiments (i.e., consumers exposed to 38 and 42°C died during acclimation, thus we added 32 and 36°C, the maximal temperature at which individuals could survive the acclimation process). Prior starvation yielded basal conditions of the organisms at culturing conditions (see Methods S.2 in Data S1). For all experiments, a temporally blocked design (*n* = 3 in each block, *n* = 6 across two blocks) ensured that replicates were tested at each temperature in two separate incubators to prevent pseudoreplication at the level of the incubator. For respiration and net photosynthesis, controls contained a 0.2 μm filtered medium. For ingestion, controls instead contained each algal species without consumers. To avoid thermal shock, each replicate (including controls) was given 1 h of ramp‐up acclimation in water baths following starvation to the assay temperature prior to experiments. Oxygen depletion for 30 min in dark conditions measured respiration. Oxygen production for 30 min in constant light conditions (PAR intensity of 67–70 μmol m^−2^ s^−1^) measured net photosynthesis immediately following the measurement of respiration in algal tests. The employed time for oxygen measurements, that is, 30 min, exceeded that of previous studies using similar devices (e.g., Schaum et al., 2017; Uiterwaal & DeLong, 2019; Warkentin et al., 2007). For respiration and net photosynthesis, replicates were 5 mL samples with no air bubbles in glass vials (SensorVial SV‐PSt5‐4mL, PreSens®) mounted on a Sensor Dish reader (PreSens®). Before tracking oxygen measurements (in mg O_2_ L^−1^), we gave the samples 1 h at assay temperatures in the incubators for stabilization of the oxygen device. For respiration and photosynthesis, consumer replicates contained five starved adult daphnids in filtered media, and algal replicates consisted of 5 mL of nutrient‐depleted algal cultures adjusted to set densities (see Methods S.2 and Figure S2 in Data S1) using a modified nutrient‐free medium for dilution. Consumer and resource respiration rates were measured separately. For ingestion, we placed five starved adult daphnids in 5 mL of highly dense algal suspension from our clonal batch cultures (either *C. reinhardtii* or *Desmodesmus* sp. taken at the exponential phase). Consumer ingestion was then measured as algal density removed from the media over 150 min in dark conditions to prevent any phototactic reaction of the daphnids, which could impact the consistency of ingestion measurements (Serra et al., 2019) as well as respiration rates. Initial algal densities did not statistically differ between control and consumer treatments within temporal blocks (all Post‐Hoc pairwise tests, *p* > .05, Methods S.4 in Data S1). We used flow cytometry (BD FACSCanto II high throughput sampler) to measure final algal densities of preserved algal samples from the algae respiration and net photosynthesis experiments, as well as at the beginning and the end of *Daphnia* ingestion experiments (see Methods S.3 in Data S1).

For physiological rates exhibiting unimodal responses to temperature (i.e., respiration and net photosynthesis), we fitted the data to a Sharpe–Schoolfield model (Schoolfield et al., 1981) described in Equation 1 which characterizes the shape of a Thermal Performance Curve (TPC), computing predictions to 0.001°C precision in the R 4.0.3 statistical environment (see Methods S.4 in Data S1):

(1) $$\text{rate}\left(T\right)=\frac{r_{\text{tref}}∙\exp^{\frac{-E_{a}}{k}\;\left(\frac{1}{T+273.15}-\frac{1}{T_{\mathrm{ref}+273.15}}\right)}}{1+\exp^{\frac{E_{h}}{k}\;\left(\frac{1}{t_{h}}-\frac{1}{T+273.15}\right)}}$$

where $\text{rate}\left(T\right)$ is the rate at a temperature *T* (°C), *k* is the Boltzmann constant with a value of 8.62e‐05, *E*_a_ is the activation energy (in electron volts, eV), that is, the slope below the optimum, *E*_h_ is the de‐activation energy (eV) at high temperature, that is the slope beyond the optimum, *t*_h_ is the temperature at which enzymes are half‐suppressed due to high temperatures, $r_{\text{tref}}$ is the rate at a standardized temperature, and *T*_ref_ is a standardization temperature (°C) (Padfield et al., 2021). These parameters are obtained from the non‐linear model (with a significance level of .05). TPCs also provide other complementary thermal performance parameters (i.e., the optimum temperature, *T*_opt_, and maximal rate at optimum temperature, *MRP*, Figure 1). We evaluated the fit of unimodal rates for the 25 curves described by Padfield et al. (2021). The selected model—the Sharpe–Schoolfield model (Equation 1)—best fit the majority of the rates and it offered the additional advantage of providing estimates of previously described TPC parameters. Bootstrapping using the case resampling method yielded confidence intervals for the TPC parameters (see Methods S.4 in Data S1). Linear responses of ingestion rates with temperature were assessed with general linear models (GLM) after verifying that both data and model residuals complied with parametric assumptions. For ingestion rates, we independently fitted the data to linear regression models for each resource species and computed predicted ingestion to 0.001°C precision. Ingestion rates of *D. pulex* feeding on *Desmodesmus* sp. showed no significant temperature dependence, hence to calculate predicted values of ingestion we fitted ingestion data to a linear regression with the slope constrained to a value of zero. Subsequently, we calculated species energetic balances as net photosynthesis‐to‐respiration ratio (*P*/*R*_r_) for resources, or as ingestion‐to‐respiration ratio (*I*/*R*_c_) for consumers, using predicted values of each per capita rate from either the TPC or GLM models. From individual energetic balances, we first identified the intraspecific thermal mismatches (intra‐TM) for each species starting at those temperatures at which the balance declined with increasing temperature. Second, we inferred interspecific thermal mismatches (inter‐TM) by comparing individual energetic balances for both consumer–resource pairs and identified the thermal mismatch regions (Figure 1), where: (1) trends of energetic balances contrast between the interacting species; and (2) both species reduce their energetic balance with increasing temperature. The inter‐TMs were used to increase our qualitative understanding of the outcomes of thermal dependencies of interaction strength. Third, we calculated the natural logarithm of the consumer–resource energetic balance (CREBs, Box 1) for each interacting pair as the ratio between the consumer energetic balance and the resource energetic balance to get qualitative predictions on the trends of interaction strength with temperature. Finally, we experimentally measured thermal dependencies of interaction strength, using the Dynamic Index, for each consumer–resource pair and verified whether our predictions using CREBs and the thermal mismatches matched the trends of interaction strengths calculated across temperatures.

### 2.2 Computing energy loss and gain in resources

Algal per capita respiration rates ($R_{r}$, mg O_2_ L^−1^ min^−1^ per cell) were calculated using Equation 2:

(2) $$\text{rate}=-1*\frac{\left(m_{t}-\bar{m_{c}}\right)}{d}$$

where for each replicate, $m_{t}$ is the slope of the linear regression between oxygen saturation and time, $\bar{m_{c}}$ is the mean slope of the controls from the same temporal block, and *d* is algae density. We multiplied the oxygen difference between algae and control replicates by −1 so that *R*_r_ describes the algal per capita oxygen consumption. Although algal batch cultures were non‐axenic, bacteria densities were negligible, and, therefore, bacterial respiration was not taken into account in our resource respiration rate measurements. Per capita rates of net photosynthesis (*P*, mg O_2_ L^−1^ min^−1^ per cell) were calculated using Equation 2, but without multiplying the oxygen difference between algae and control replicates by −1.

### 2.3 Computing energy loss and gain in the consumer

Per capita rate of consumer respiration (*R*_c_, mg O_2_ L^−1^ min^−1^ per *Daphnia*) was calculated using Equation 2 but using $m_{t}$ as the slope of oxygen depletion for replicates containing daphnids, and *d* as the number of daphnids alive at the beginning of the experiment in *Daphnia* replicates instead. The value of *d* was generally 5, but in exceptional cases, individuals died during the acclimation process. We converted consumer per capita respiration rates (*R*_c_, mg O_2_ L^−1^ min^−1^ per *Daphnia*) into energetic equivalents of metabolism ($R_{C}^{E}$, J L^−1^ h^−1^ per *Daphnia*) by assuming that 1 mg O_2_ equals 14.06 J (Peters, 1983) (see Methods S.5 in Data S1). Per capita rates of raw ingestion (rI, cells mL^−1^ h^−1^ per *Daphnia*) were calculated using Equation 3:

(3) $$\mathrm{rI}=\frac{\left(D_{T0}-D_{T150}\right)+\left(\bar{C_{T150}-C_{T0}}\right)}{\mathrm{yt}}$$

where $D_{T0}$ and $D_{T150}$ are resource densities in *Daphnia* treatment replicates at the beginning and the end of the experiment, $\bar{C_{T0}}$ and $\bar{C_{T150}}$ are the mean value of resource densities at the beginning and the end of the experiment across control replicates (without consumers) from each temporal block (*n* = 3 × 2 temporal blocks), y is the number of *consumers* in the *Daphnia* treatment replicates alive at the beginning of the experiment, and t is the length of the experiment (150 min). Resource densities were at high concentrations (~4.5 × 10^5^ cells mL^−1^ for *C. reinhardtii* and ~1.3 × 10^6^ cells mL^−1^ *Desmodesmus* sp. based on pilot experiments) to achieve saturating values of the functional response (West & Post, 2016). Similar to consumer respiration rates, per capita raw ingestion rates (rI, cells mL^−1^ h^−1^ per *Daphnia*) were transformed into energy equivalents (${\mathrm{rI}}^{E}$, J L^−1^ h^−1^ per *Daphnia*) to make them quantitatively comparable (see Methods S.5 in Data S1). To account for imperfect assimilation, we then calculated final per capita ingestion rates, $I^{E}$ J L^−1^ h^−1^ per *Daphnia*, by multiplying per capita raw ingestion rates in energy units, ${\mathrm{rI}}^{E}$, by a unitless assimilation efficiency for each algal species. Assimilation efficiencies were assumed to be constant across temperatures set at 14% when *D. pulex* fed on *C. reinhardtii* and 10% when it instead fed on *Desmodesmus* sp. (see Methods S.5 in Data S1). Adult experimental daphnids averaged 2.08 ± 0.1 mm in length (mean ± SD).

### 2.4 Consumer–resource interaction strength

From the ingestion experiments, using algal densities at the end of the tests (i.e., *T*_150_), we next estimated per capita interaction strength (IS) for each consumer–resource pair as the log‐response ratio, also called the Dynamic Index (Berlow et al., 1999), but using the more intuitive inverse form (as in Schaum et al., 2018):

(4) $$\text{IS}=\frac{\ln\left(\frac{N}{D}\right)}{\mathrm{yt}}$$

where *N* is the algal densities without consumers present (mean value of three control replicates per temporal block), *D* is the algal densities with consumers present (*Daphnia* treatment replicates), *y* is the number of consumers in each *Daphnia* treatment replicates and *t* is the experiment length. The Dynamic Index does not depend on equilibrium conditions and, therefore, works well for short‐term experiments (Berlow et al., 2004; Laska & Wootton, 1998). Here the index measures the absolute value of per capita interaction strength by time, accounting for algal growth with and without consumers (O'Connor, 2009). A large value of IS indicates a strong per capita impact of the consumer on the resource density. Conversely, low values of IS indicate a weak impact of one consumer on resource densities. We fitted values of interaction strength to general linear models (GLM) by conducting a full linear regression including the interaction between temperature and algal species and performed a two‐way analysis of variance (ANOVA) to get slopes and summary statistics (see Methods S.4 in Data S1).

## 3 RESULTS

### 3.1 Intraspecific thermal mismatches in resources

**FIGURE 2:**
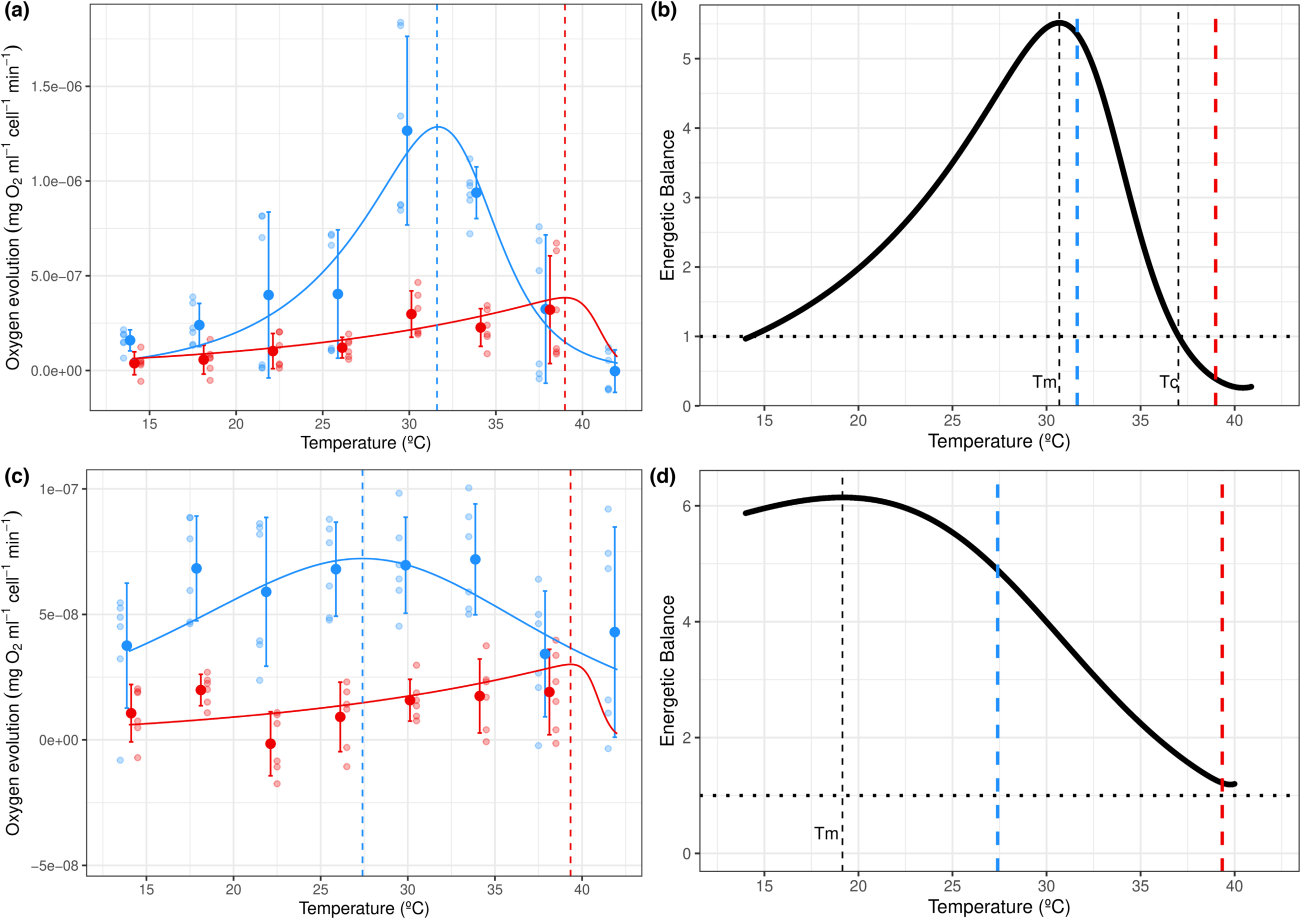
Thermal performance curves (TPCs) of energy gain and loss and energetic balance for *Chlamydomonas reinhardtii* (a and b) and *Desmodesmus* sp. (c and d). In (a) and (c), red corresponds to per capita respiration rates, and blue to per capita net photosynthesis rates. TPC trends depict calculated predicted values using a nonlinear mixed effect model fitted to the parameter data using the Sharpe–Schoolfield equation model. Bold points represent mean values with error bars showing ± SEM (*n* = 6). Light points denote raw values. In (b) and (d), the energetic balance is computed as the net photosynthesis (*P*) to respiration (*R*_r_) ratio (*P*/*R*_r_) using predicted values of each rate obtained from models. Vertical black‐dashed lines correspond to temperatures at which the maximal value of the energetic balance is obtained (*T*_m_ = 30.7°C for *C. reinhardtii* and *T*_m_ = 19.15°C for *Desmodesmus* sp.) and the crossover temperature (*T*_c_ = 37.01°C only found for *C. reinhardtii*) when the *P*/*R*_r_ ratio equals one. Intraspecific thermal mismatches are expected for temperatures above *T*_m_. In (a) and (b), vertical‐colored dashed lines depict optimum temperatures of net photosynthesis in blue ($T_{\mathrm{opt}}^{P}$ = 31.6°C for *C. reinhardtii* and $T_{\mathrm{opt}}^{P}$ = 27.40°C for *Desmodesmus* sp.) and respiration in red ($T_{\mathrm{opt}}^{R}$ = 39°C for *C. reinhardtii* and $T_{\mathrm{opt}}^{R}$ = 39.34°C for *Desmodesmus* sp.).

Thermal performance of energy gain (i.e., photosynthesis) and loss (i.e., respiration) differed within and across resources (Table 1, Tables S1 and S2, Figure 2). Photosynthesis was more sensitive to temperature increase than respiration for both resource species, with almost a 4‐fold increase for *C. reinhardtii* for temperatures below the optimum of the G/L ratio (*T*_m_). For both resources, optimum temperatures were higher for respiration than for net photosynthesis ($T_{\mathrm{opt}}^{R}\;>\;T_{\mathrm{opt}}^{P}$, see Table 1). Although respiration temperature optima were very similar between algae species, optimum of net photosynthesis was about 4 degrees higher for *C. reinhardtii*. These differential temperature sensitivities and optima for energy gain and loss led to important intraspecific thermal mismatches, with marked differences between species (Figure 2b,d). Thus, although both resources exhibited unimodal trends of energetic balances, intraspecific thermal mismatches were more pronounced for *Desmodesmus* sp. than for *C. reinhardtii* as they arose at lower temperatures. While the crossover temperature, *T*_c_ (i.e., G/L ratio = 1) was not identified for *Desmodesmus* sp. within the temperature range tested here, this value occurred at a relatively high temperature for *C. reinhardtii*, near the optimum of respiration.

**TABLE 1:** Estimated parameters of thermal performance curves of resources and consumer per capita rates that followed unimodal responses with temperature.

Species	Rates	*E*_a_ (eV)	*T*_opt_ (°C)	MRP ng O_2_ L^−1^ min^−1^ per cell	MRP pg O_2_ min^−1^ per cell	*E*_h_ (eV)
*C. reinhardtii*	Net photosynthesis (*P*)	1.44	31.66	1.29	6.45	5.27
	Respiration (*R*_r_)	0.57	38.99	0.384	1.92	13.73
*Desmodesmus* sp.	Net photosynthesis (*P*)	0.66	27.40	0.072	0.36	1.66
	Respiration (*R*_r_)	0.50	39.34	0.003	0.015	20
*D. pulex*	Respiration (*R*_c_)	1.08	29.40	2.00*	0.01**	1.99

*Note*: Sharpe–Schoolfield model parameters show mean estimated values of activation energy (*E*_a_), deactivation energy (*E*_h_), optimum temperature (*T*_opt_), and maximum rate of performance (MRP) for resources' net photosynthesis (*P*) and respiration (*R*_r_) rates, and consumer respiration (*R*_c_) rates.

* *D. pulex* respiration units are J per *Daphnia* L^−1^ h^−1^

** *D. pulex* respiration units' volume corrected are J per *Daphnia* h^−1^.

### 3.2 Intraspecific thermal mismatches in the consumer

**FIGURE 3:**
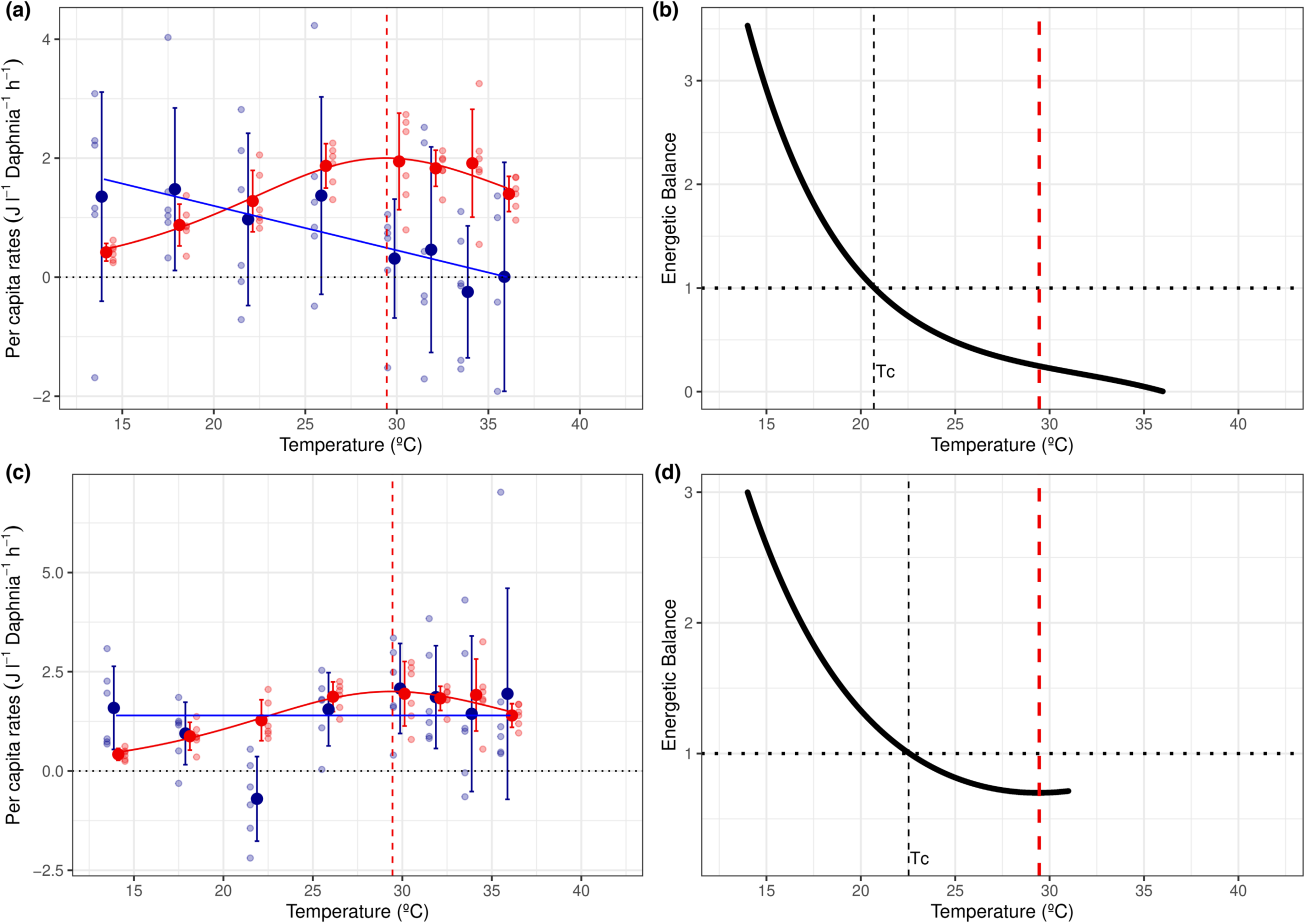
Thermal Performance Curves (TPCs) of energy gain and loss and energetic efficiencies for the consumer *D. pulex* feeding on either *C. reinhardtii* (a and b) or *Desmodesmus* sp. (c and d). In (a) and (c), red corresponds to energy loss (per capita respiration rates, *R*_c_), and blue to energy gain (per capita ingestion rates, *I* ^E^) both transformed into energy equivalents (J L^−1^ per *Daphnia* h^−1^). The TPC of consumer respiration depicts calculated predicted values using the Sharpe–Schoolfield equation model, and ingestion rates depict calculated predicted values for each resource species using GLMs. Bold data points represent mean values with error bars showing ± SEM (*n* = 6 replicates per tested temperature for each parameter, except for ingestion at 36°C with *n* = 4 C for *C. reinhardtii* due to outliers). Light points denote raw values. In (b) and (d), the energetic balance is calculated as the ingestion (*I*) to respiration (*R*_c_) ratio of *D. pulex* feeding on *C. reinhardtii* (b) or *Desmodesmus* sp. (d). The horizontal black dotted line indicates a value of *I*/*R*_c_ ratio equal to one, where energy gain equals loss. Energetic balances show intraspecific thermal mismatches for *Daphnia* with increasing temperature for the whole thermal range tested when feeding on either resource species. Vertical black‐dashed lines show critical temperatures (*T*_c_; *I* ^E^/*R*_c_ ratio = 1) occurring at 20.7°C when *D. pulex* feeds on *C. reinhardtii* and at 22.5°C when it feeds on *Desmodesmus* sp. Vertical dashed lines in red depict the optimum temperature of *D. pulex* respiration rates ($T_{\mathrm{opt}}^{R}$ = 29.45°C).

The consumer showed marked differences between thermal sensitivities of energy gain (i.e., ingestion) and loss (i.e., respiration) (Table 1, Tables S1, Figure 3). Consumer respiration was unimodal with temperature. However, thermal dependencies of ingestion showed linear and contrasting responses depending on what algal species served as a food source. While ingestion declined slowly with temperature when consumers fed on *C. reinhardtii* (*β* = −.075, SE = .02, *R* ^2^ = 0.15, *F*_1,44_ = 7.573, *p* = .009), ingestion was temperature‐independent when consumers fed on *Desmodesmus* sp. (*β* = .05, SE = .03, *R* ^2^ = 0.05, *F*_1,45_ = 2.655, *p* = .11). Consumer respiration (*R*_c_) was more sensitive to increases in temperature than ingestion (*I*) feeding on either resource species. These thermal differences led to declines in the energetic balances for the consumer and yielded intraspecific thermal mismatches, for the whole temperature range tested (Figure 3b,d). The consumer's crossover temperatures (*T*_c_) differed slightly between resource species, with a higher value when it fed on *Desmodesmus* sp.

### 3.3 Interspecific thermal mismatches

Based on the observed intraspecific thermal mismatches at the individual level (Figures 2 and 3, Figure S2), we expected interspecific thermal mismatches for both consumer–resource pairs to emerge at low temperatures, 14°C. However, differences in thermal regions ‘1’ and ‘2’ of the interspecific thermal mismatch (Figure 1) between both interacting pairs points to a difference in the nature of the thermal mismatch and the implications for the interaction. For the *Daphnia*—*C. reinhardtii* pair, interspecific thermal mismatches arose at 14°C (Figure S2), as increases in temperature reduced the energy balance of the consumer (Figure 3b) but increased that of the resource (Figure 2a). Thus, the thermal region ‘1’ is defined between 14 and 30.7°C. Temperatures beyond this thermal region (>30.7°C) caused declines in energetic balances for both interacting species (i.e., thermal region ‘2’ of inter‐TM). Alternatively, for the *Daphnia*–*Desmodesmus* sp. pair, interspecific thermal mismatches also arose at 14°C. However, for this pair thermal region ‘1’ of the inter‐TM occurred between 14 and 19°C, and therefore we observed that further increases in temperature generally decreased the energetic balance for both interacting species (>19°C, thermal region ‘2’).

### 3.4 Consumer–resource energetic balances of interacting species

**FIGURE 4:**
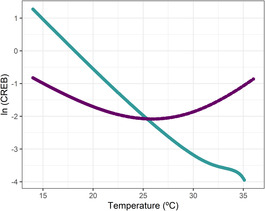
Consumer–Resource Energetic Balances (CREBs) of consumer *D. pulex* feeding either on *C. reinhardtii* (in blue‐green) or *Desmodesmus* sp. (in purple) across temperatures. CREBs are calculated by dividing the consumer energetic balance (i.e., ratio between energy gain and loss) by the resource‐energetic balance. Trends of CREBs with temperature are used to predict the thermal response of interaction strength for the interacting species: a negative trend with temperature would translate into a declining pattern for interaction strength and vice versa (see Box 1).

Consumer–Resource Energetic Balances (CREBs) yielded contrasting patterns between the two interacting consumer–resource pairs (Figure 4). CREB declined steadily with warming for the *D. pulex*–*C. reinhardtii* interaction while producing a U‐shaped response for the *D. pulex*–*Desmodesmus* pair. Based on these results we predicted a declining trend for the interaction strength between *D. pulex* and *C. reinhardtii* and either a U‐shape or a constant trend for the interaction strength between *D. pulex* and *Desmodesmus* sp.

### 3.5 Thermal dependencies of interaction strengths

**FIGURE 5:**
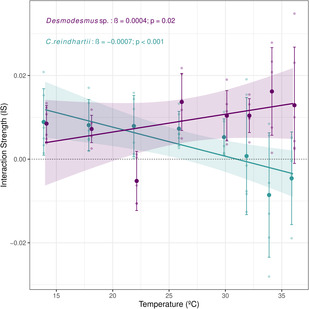
Effects of temperature on hourly per capita Interaction strength (IS). Interaction strengths are computed as the log response ratio (i.e., the Dynamic Index) of *D. pulex* feeding either on *Desmodesmus* sp. (in purple) or on *C. reinhardtii* (in turquoise). Bold data points represent mean values of IS with error bars showing ± SEM (*n* = 6, except for *C. reinhardtii* at 36°C and *Desmodesmus* sp. at 22°C where IS values had five replicates instead due to the presence of an outlier). Light points denote raw values. Beta values (*β*) represent the slope of the linear regression between temperature and IS for each resource, showing the significance value for each regression (*p*) (see Tables S4 and S5 and Methods S.4 in Data S1 for statistical details).

The effect of temperature on interaction strength showed opposite trends for the two consumer–resource interactions (ANOVA test: *F*_1,90_ = 20.24, *p* < .001; Tables S4 and S5, Figure 5). Temperature increased interaction strength when consumers fed on *Desmodesmus* sp. (*β* = .0004, *p* = .02), while interaction strength decreased with temperature when the consumer fed on *C. reinhardtii* (*β* = −.0007, *p* < .001). The differences in thermal regions of interspecific thermal mismatches between species pairs could explain the differences in thermal dependencies of interaction strength. Consumers feeding on *C. reinhardtii* suffered from energetic deficits with increasing temperatures while the resource's energetic balance increased for the majority of the thermal gradient (<30.7°C, thermal region ‘1’). This consumer intraspecific energetic mismatch in combination with the resource energetic gains could reduce the intensity of the per capita effect of the consumer on *C. reinhardtii* with warming (Tables S4 and S5, Figures 2b and 3b, Figure S1). Consumers feeding on *Desmodesmus* sp. suffered from energetic deficits throughout the temperature gradient (Figure 3d). However, the resource energetic balance also decreased for most temperatures (Figure 2d); only at low temperatures did the energetic balance of *Desmodesmus* sp. increase (14–19°C). Thus, the interspecific thermal mismatch (thermal region 1) was restricted to only this narrow temperature range for this interaction pair. Nevertheless, the consumer maintained constant ingestion rates (Figure 3c) which could translate into either a constant or enhanced intensity of the per capita effect of the consumer on *Desmodesmus* sp. with rising temperatures. In addition, we observed that the consumer intraspecific thermal mismatch was not as strong when feeding on *Desmodesmus* sp. (i.e., crossover temperature, *T*_c_, was slightly larger for *Desmodesmus* sp.) compared to when it fed on *C. reinhardtii* instead (Figure 3b,d). Thus, we consider that the net effect of the consumer on the resource increased with temperature which is reflected in the thermal dependence of interaction strength (Figure 5).

## 4 DISCUSSION

Trophic interactions, which in certain cases are strongly responsive to temperature change, constitute the building blocks of ecological communities. Therefore, understanding how temperature affects consumers, resources, and their interactions is necessary for predicting the impacts of climate change on ecosystems. We performed experiments to determine the thermal dependencies of energy gain and loss of two consumer–resource pairs. This allowed us to examine the energetic balance of species and the intra‐ and inter‐specific thermal mismatches—that is, the negative effects of warming on species’ energetic balances (Figure 1)—which can determine species' performance and survival. We hypothesized that consumer–resource energetic balances, that is, the ratio of consumer's to resource's energetic balances, would predict how interaction strength changes with increasing temperatures. The novelty of our approach is twofold. First, we simultaneously considered the energetic traits of both the resource and the consumer. Second, we show how energetic constraints of interacting individuals mediated by temperature change allow for predicting interaction strength between consumer and resource populations. This approach provides a new mechanistic understanding of the impacts of climatic warming on trophic interactions by establishing new links between thermal ecology, focused on thermal dependencies of energetic traits, and food web ecology centered on measuring interaction strengths.

### 4.1 Does the metabolic status of resources matter?

Intraspecific thermal mismatches depend on two main factors that determine how the balance of energy gain and loss changes as temperature increases: the relative sensitivities to temperature increase (i.e., slopes) and the optimal temperatures. Compared to previously reported values, our estimated slopes for the resource species (i.e., activation energies *Ea*, Table 1, Tables S1 and S2) were higher for photosynthesis (Barton et al., 2020; Lopez‐Urrutia et al., 2006; Padfield et al., 2016) and lower for respiration (Barton et al., 2020). Hence, we observed that energy gain (i.e., net photosynthesis) increased faster than energy loss (i.e., respiration) with warming. This contradicts the well‐supported result that respiration has a greater thermal sensitivity than photosynthesis in autotrophs (Allen et al., 2005; Anderson‐Teixeira et al., 2011; Lopez‐Urrutia et al., 2006; Padfield et al., 2016). This divergence may be explained by differences in the metabolic state of the resource (Marañón et al., 2018; Thomas et al., 2017). In our case, resources were at basal metabolic conditions following prolonged starvation (i.e., nutrient‐depleted conditions). These experimental conditions stand in contrast to most studies where species grow in nutrient‐rich media (e.g., Barton et al., 2020; Padfield et al., 2016), often exceeding the nutrient availability observed in nature. Given the variety of conditions observed in natural communities, it remains unclear which approach ‐starvation or not‐ best fits natural conditions. Nonetheless, our experiment best mimics the effects of temperature on autotrophs in cases where nutrients are low (e.g., oligotrophic). Although some respiration rates for *Desmodesmus* sp. showed negative values, we believe that they were likely caused by the small signal relative to the instrument sensitivity of this algal species, that is, low photosynthesis and respiration values, compared to those for *C. reinhardtii* and the correction using control replicates (i.e., mean value of oxygen across control replicates from the same temporal block exceeded that of the algae replicate). Even though using a higher density of this algae could have prevented these issues, we believe that the trends in both respiration and photosynthesis with temperature were clear and consistent for *Desmodesmus* sp.

### 4.2 Consumer's intraspecific thermal mismatches

For the consumer, our results of thermal sensitivities (Table 1, Tables S1 and S2) agree with previous observations: respiration was more sensitive to temperature than ingestion (Archer et al., 2019; Rall et al., 2010; Vucic‐Pestic et al., 2011), resulting in intraspecific thermal mismatches. Although ingestion rates generally increase with temperature, we found no significant thermal dependence when *Daphnia* fed on *Desmodesmus* sp. (similar to Rall et al., 2010; Iles, 2014; Archer et al., 2019) and a negative trend when it fed on *C. reinhardtii*. As energy acquisition for the consumer had a weak relationship with temperature, this translated into lower values of crossover temperatures (*T*_c_; 20.7°C for *C. reinhardtii*, 22.5°C for *Desmodesmus* sp.) than expected for *D. pulex* (Goss & Bunting, 1983). Explanations for the low *T*_c_ could be the difference in the metabolic state of the consumers (Brown et al., 2004; Clarke & Fraser, 2004)—our daphnids were at resting metabolic conditions—and the length of the exposure to temperature. Exceeding these crossover temperatures under short‐term exposures causes energy deficits and stress for the consumers, though this does not necessarily translate into mortality. Ways to buffer against this stress in the longer term could include a reduction in body size to reduce metabolic demands (Khan & Khan, 2008; Van Doorslaer et al., 2010). Ultimately, however, if the organism cannot respond, extinctions would be expected. By conducting the *D. pulex* ingestion experiments in darkness, we were able to best mimic natural conditions. Daphnids generally react to predators with diel vertical migration. During the day they remain at lower depths, minimizing predation. They migrate up to the surface at night, exhibiting greater feeding activity under the cover of darkness (Serra et al., 2019).

### 4.3 Are changes of interaction strength with temperature context‐dependent?

Our definition of consumer–resource interspecific thermal mismatches is based on the comparison of energetic balances and intraspecific thermal mismatches of resources and consumers. Interspecific thermal mismatches combined with the calculation of the consumer–resource energetic balances (CREBs) can help to understand and predict how interaction strength changes with temperature. Although interspecific thermal mismatches for both consumer–resource pairs covered the full temperature range, the nature and implications of the mismatch differed for both species pairs. This can be better explained by examining the contrasting trends for the two CREBs (Figure 4).

For the *Daphnia*–*C. reinhardtii* interaction, the picture is clear. A wide range for the thermal region 1 of their interspecific thermal mismatch corresponded to a decreasing CREB with temperature, which indicates that the consumer energetic balance decreased faster relative to that of the resource with warming (Figures 2b and 3b, Figure S2). Thus, interaction strength should also decrease (Figure 5). In other words, if decreasing CREB coincides with decreasing ingestion rates, consumer metabolic demands will need to be met through other means, such as reduced energy expenditure or decreased body size (Khan & Khan, 2008; Van Doorslaer et al., 2010). Despite a decrease in resource energy reserves, this should weaken the interaction strength.

For the *Daphnia*–*Desmodesmus* sp. interaction, both species suffered from intraspecific mismatches for most temperatures—that is, a wide thermal region 2 for the interspecific mismatch—(Figures 2d and 3d, Figure S2) and the CREB was U‐shaped (Figure 4). This suggests that above a certain temperature, the consumer energetic balance decreased slower than the resource energetic balance, which could mean that the pressure (i.e., interaction strength) exerted upon the resource should increase with warming. This is where CREB can prove very useful, as it identifies which of the two species suffers more strongly (in terms of its energetic balance) with warming. Increasing or constant CREB should translate into increased interaction strength, which is in line with the trend observed for this pair (Figure 5).

This divergence highlights a strong context‐dependence on the effects of consumers on resource population densities across temperature gradients. Such context‐dependence is not surprising (Betini et al., 2019; Uiterwaal & DeLong, 2020). Differences in thermal optima for the two resource species' energetic balances may explain this divergence between experiments (Betini et al., 2019). Some authors showed that interaction strength increases with temperature, others that it decreases, and others that it has a hump‐shaped relationship (Amarasekare, 2015; Fussmann et al., 2014; Gilbert et al., 2014; Rall et al., 2010; Synodinos et al., 2021; Vucic‐Pestic et al., 2011).

Even though the interaction strength results may be context‐dependent, our mechanistic interpretation based on energetic mismatches is not. Interspecific thermal mismatches and the calculation of CREBs consider both resource and consumer performance independently and simultaneously to predict the effects of warming on the interaction of the two species.

## AUTHOR CONTRIBUTIONS

**Soraya Álvarez‐Codesal:** Conceptualization (equal); formal analysis (lead); investigation (lead); methodology (lead); writing – original draft (lead). **Cara A. Faillace:** Conceptualization (equal); methodology (supporting); supervision (equal); writing – review and editing (equal). **Alexandre Garreau:** Investigation (supporting); methodology (equal). **Elvire Bestion:** Formal analysis (supporting); writing – review and editing (equal). **Alexis D. Synodinos:** Writing – review and editing (equal). **José M. Montoya:** Conceptualization (lead); funding acquisition (lead); supervision (lead); writing – review and editing (equal).

## Supporting information


Data S1.

## Data Availability

The data that support the findings of this study are openly available in zenodo at https://zenodo.org/record/7974614. DOI: 10.5281/zenodo.7974614
