# Supplementary material for: Thermal mismatches explain consumer–resource dynamics in response to environmental warming
Source: Ecol Evol. 2023 Jun 13;13(6):e10179. doi: 10.1002/ece3.10179 (PMC10264966; doi:10.1002/ece3.10179)
Supplement: Supplementary file 1 — Data S1. [file ECE3-13-e10179-s001.docx]

**SUPPORTING INFORMATION**

**Supplementary figures and tables**


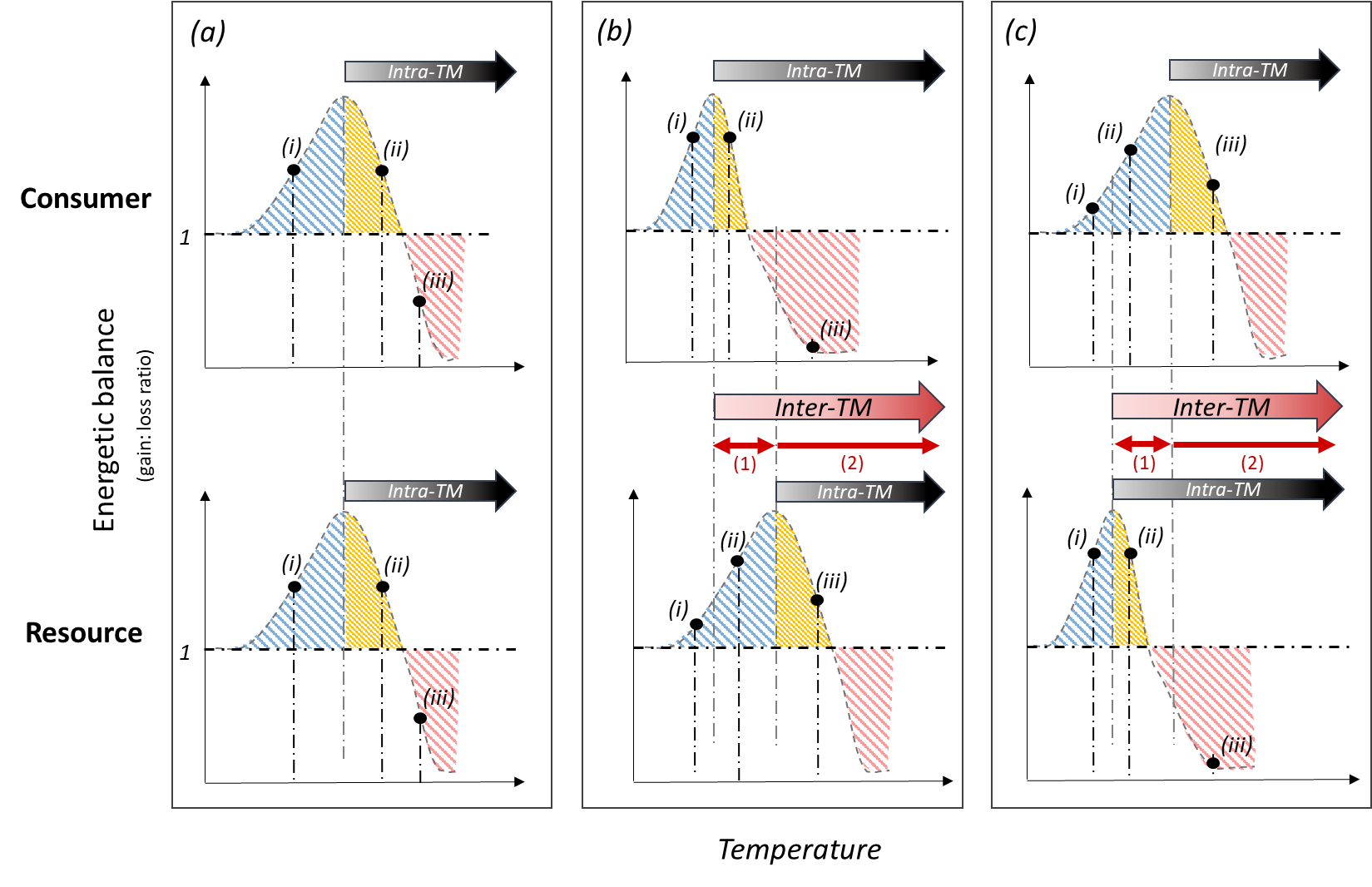


**Figure S1. Possible scenarios of interspecific thermal mismatches (Inter-TM) in a consumer-resource interaction.** Energetic balances (i.e. gain-to-loss ratios) on the top represent those of the consumer and on the bottom, those of the resource. Coloured shaded-regions depict energetic balance trends with increasing temperatures, in blue rising trends, and in orange and red declining trends over and below unity respectively. Scenario (a) depicts no inter-TM while b) and c) show cases of inter-TM. In (a) the thermal dependency of consumer energetic balance matches that of the resource, so no mismatch in the interaction is expected. At low temperature (i), increases of temperature lead to increases of energy availability for both organisms. Then, at intermediate temperatures (ii), increases of temperature reduce the energy availability for both organisms, causing intraspecific thermal mismatches (Intra-TM) in both species. Thus, both organisms suffer from energy deficits in the same way with temperature. Finally, at high temperatures (iii), the ratio falls below 1 for both species, meaning that the energy gain cannot compensate for the energy loss, so energy deficits will be strong for both organisms. In (b) thermal dependency of the consumer energetic balance differs from that of the resource, so inter-TMs are expected with the consumer affected from energy deficits at lower temperatures than the resource. At low temperature (i), increases of temperature translate into an increase of energy availability for both organisms. Then, at intermediate temperatures (ii), increases of temperature reduce the energy available to the consumer, but not the resource, which still increases. This causes an inter-TM because the consumer suffers from energy deficits while the energy available to the resource still increases with temperature. At high temperatures (iii), the consumer struggles to cope with energy losses as the ratio falls below 1, while the resource species still maintains an energy balance over unity. However, increases of temperature in this thermal region will reduce the energy available for resource species. In (c) similarly to (b), thermal dependency of the energetic balance of consumer differs from that of the resource, so an inter-TM is expected, but the resource suffers from energy deficits at lower temperatures compared to the consumer. Predicted temperature scenarios (i, ii, iii) of case (c) show the opposite situation compared to those in scenario (b), with the resource instead suffering from energetic deficits at lower temperatures than does the consumer. In nature, scenarios a) and b) are most likely to happen for ectothermic species, while case c) may occur hypothetically in a situation where an endotherm feeds on a thermally sensitive ectotherm.


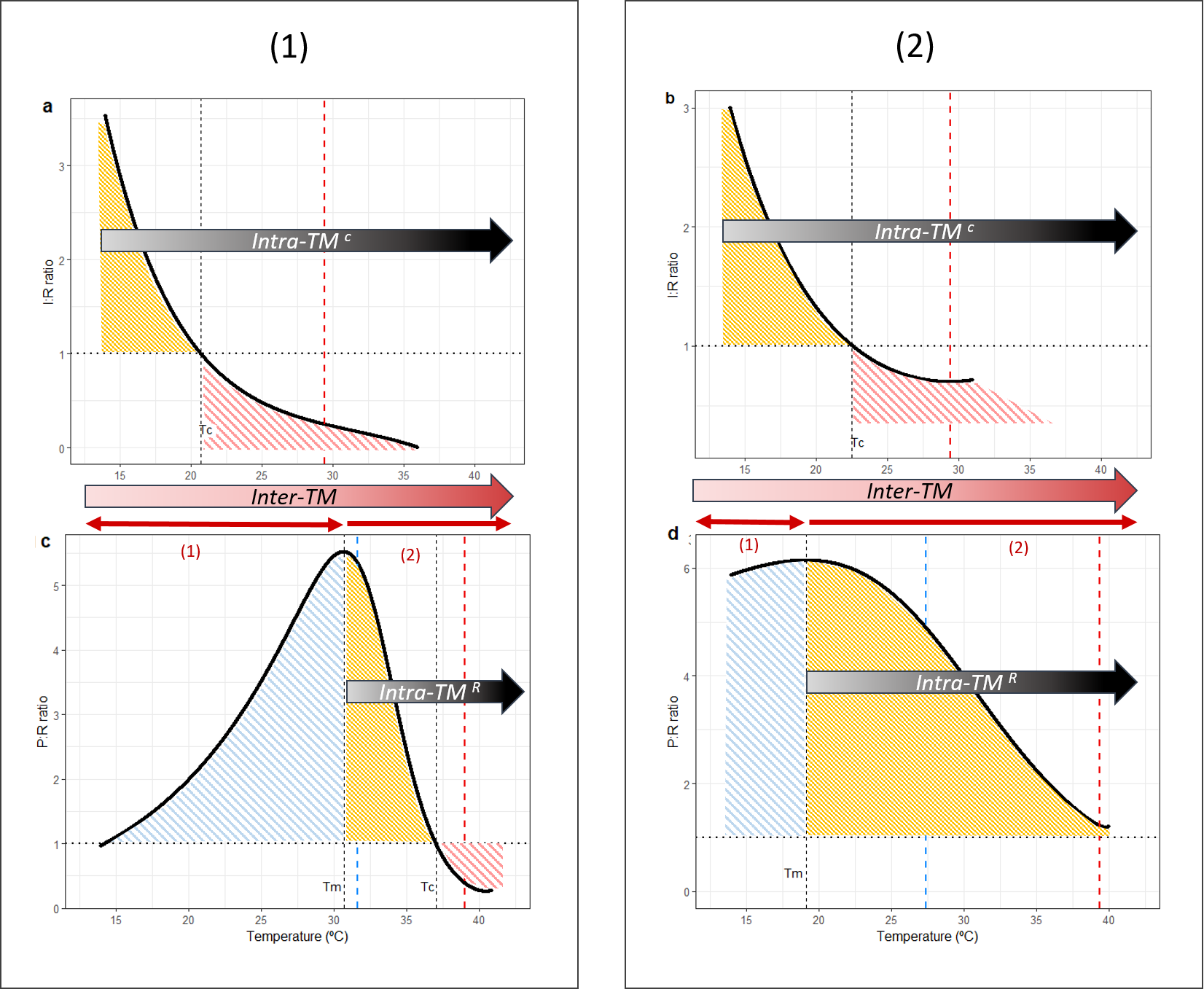


**Figure S2. Consumer and resource species’ thermal mismatches based on species energetic balances from results.** Energetic balances (i.e. gain-to-loss ratios) are calculated from predicted values of energy gain and loss parameters. On the top represent those of the consumer, calculated as the ingestion-to-respiration ratio, feeding either on *Chlamydomonas reinhardtii* (1-a) or Desmodesmus sp. (2-b). On the bottom, energetic balances of resource species (*Chlamydomonas reinhardtii* 1-c, *Desmodesmus sp*. 2-d) are calculated as net photosynthesis-to-respiration ratio. Coloured regions depict energetic balance trends with increasing temperatures. In blue rising trends, and in orange and red declining trends over and below unity respectively. Black arrows show species’ intraspecific thermal mismatches, i.e., negative effects on species energetic balances due to warming. Red arrows show interspecific thermal mismatches between interacting species with two distinguishable thermal regions, where: 1) trends of energetic balances contrast between the interacting species; and 2) both species reduce their energetic balance with increasing temperature. Vertical coloured-dashed lines in black depict the temperature at which the G/L ratio is maximized (Tm) and the crossover temperature (Tc) where energy gain equals energy loss (ratio = 1). In blue and red, vertical-dashed lines depict optimum temperatures for net photosynthesis and respiration respectively.

**Table S1. Sharpe-Schoolfield parameter estimates with associated standard errors (SE), t-values, residual standard error, degrees of freedom (DF) and number of iterations to convergence from models describing the response of respiration and net photosynthesis rates with temperature for *C. reinhardtii* and *Desmodesmus* sp., and respiration rates for *D. pulex***. E_a_ is activation energy (eV), E_h_ is high temperature de-activation energy (eV), T_h_ is the temperature at which enzyme is ½ active and ½ suppressed due to high temperatures (⁰C) and T_ref_ is standardisation temperature at which rates are not inactivated by high temperatures.

| **Species** | **Rate** | **Parameter** | **Estimate** | **SE** | **t-value** | **p-value** | **Residual standard error** | **DF** | **Num. convergence** |
| --- | --- | --- | --- | --- | --- | --- | --- | --- | --- |
| ***C. reinhardtii*** | Respiration | *T*_ref_ | 6.86e-08 | 2.76e-08 | 2.48 | < 0.01 | 1.343e-07 | 44 | 41 |
|  |  | E_a_ | 0.57 | 0.20 | 2.91 | < 0.01 |  |  |  |
|  |  | E_h_ | 13.74 | 69.54 | 0.20 | 0.84 |  |  |  |
|  |  | T_h_ | 40.92 | 5.66 | 7.22 | < 0.001 |  |  |  |
|  | Net photosynthesis | *T*_ref_ | 7.49e-08 | 4.21e-08 | 1.78 | 0.082 | 3.014e-07 | 44 | 50 |
|  |  | E_a_ | 1.44 | 0.34 | 4.27 | < 0.001 |  |  |  |
|  |  | E_h_ | 5.27 | 1.17 | 4.48 | < 0.001 |  |  |  |
|  |  | T_h_ | 33.16 | 0.99 | 33.39 | < 0.001 |  |  |  |
| ***Desmodesmus sp.*** | Respiration | *T*_ref_ | 6.446e-09 | 3.378e-09 | 1.91 | 0.06 | 1.557e-08 | 44 | 52 |
|  |  | E_a_ | 0.50 | 0.26 | 1.91 | 0.06 |  |  |  |
|  |  | E_h_ | 20.00 | 827.9 | 0.02 | 0.98 |  |  |  |
|  |  | T_h_ | 40.89 | 45.82 | 0.89 | 0.37 |  |  |  |
|  | Net photosynthesis | *T*_ref_ | 3.998e-08 | 8.059e-09 | 4.961 | < 0.001 |  | 44 |  |
|  |  | E_a_ | 0.66 | 0.44 | 1.49 | 0.14 | 2.587e-08 |  | 59 |
|  |  | E_h_ | 1.66 | 0.34 | 4.83 | < 0.001 |  |  |  |
|  |  | T_h_ | 29.38 | 7.75 | 3.79 | < 0.001 |  |  |  |
| ***D. pulex*** | Respiration | *T*_ref_ | 0.655 | 1.681e-04 | 3.899 | < 0.001 | 0.0005772 | 44 | 20 |
|  |  | E_a_ | 1.078 | 0.457 | 2.356 | 0.02 |  |  |  |
|  |  | E_h_ | 1.993 | 0.3605 | 5.527 | < 0.001 |  |  |  |
|  |  | T_h_ | 28.74 | 5.403 | 5.320 | < 0.001 |  |  |  |

**Table S2. Summary table of estimated Thermal Performance Curve’s (TPC) parameters and confidence intervals of resource and consumer rates.** Parameters show mean values of the activation energy (E_a_), the optimum temperature (T_opt_), the maximum rate of performance (MRP) (i.e., at optimum temperature), and the deactivation energy (E_h_) of those rates showing unimodal trends with temperature. Values in brackets show 95% confidence intervals (ci) for each parameter obtained from case bootstrapping. Significance codes from the nonlinear regression Sharpe-Schoolfield model parameters (Ea, E_h_) are: ‘***’ 0.001 ‘**’ 0.01 ‘*’ 0.05. ‡. *D. pulex* respiration units are Joules *Daphnia*^-1^ L^-1^ h^-1^).

| **Rate** | **Species** | **E_a_ [ci]**  **(eV)** | **T_opt_ [ci]**  **(⁰C)** | **MRP**  **ng O_2_ l^-1^ min^-1^ cell ^-1^** | **E_h_ [ci]**  **(eV)** |
| --- | --- | --- | --- | --- | --- |
| **Respiration** | *C. reinhardtii* | 0.57**  [0.28 – 0.74] | 38.99  [32.40 – 40.54] | 0.384  [0.26 – 0.72] | 13.73  [2.82 - 20] |
|  | *Desmodesmus sp.* | 0.50*  [-0.03 – 2.23 ] | 39.34  [19.23– 40.44] | 0.003  [0.002 – 0.004] | 20  NA |
|  | *D. pulex* | 1.08*  [0.44 – 2.22] | 29.40  [26.44 – 34.50] | 2.00 ‡  [1.62 – 2.24] | 1.99***  [1.30 – 2.58] |
| **Net photosynthesis** | *C. reinhardtii* | 1.43***  [0.76 – 4.70] | 31.66  [30.55 – 32.95] | 1.29  [0.94 – 1.74) | 5.27***  [3.46 – 7.70] |
|  | *Desmodesmus sp.* | 0.66  [0.17 – 10] | 27.40  [17.99 –32.96] | 0.072  [0.057 – 0.082] | 1.66***  [0.67 – 2.13] |

**Table S3. Summary table of thermal sensitivities of *D. pulex* ingestion.** Linear model outputs for the results presented in Fig. 4 testing the effect of temperature on ingestion rates for *D. pulex* feeding on each algal species in the form of *lm(ingestion ~ Temperature)*. For the model for *C. reinhardtii*, F_1,44_ = 7.573, while for that of *Desmodesmus* sp. F_1,45_ = 1.187. Highlighted estimates of p-values represent significance levels lower than 0.05.

| **Algal species** | **Parameter** | **Estimate** | **SE** | **t-value** | **p-value** | **R2** |
| --- | --- | --- | --- | --- | --- | --- |
| *C. reinhardtii* | Intercept | 2.69259 | 0.73497 | 3.664 | **0.0007** | 0.15 |
|  | Slope | 0.07468 | 0.02714 | -2.752 | **0.009** |  |
| *Desmodesmus sp.* | Intercept | 0.46700 | 0.71580 | 0.652 | 0.517 | 0.03 |
|  | Slope | 0.02856 | 0.02622 | 1.089 | 0.282 |  |

**Table S4.** **Statistical results of ANOVA for the effects of temperature and algae species on interaction strength in a short-term experiment with *Daphnia pulex* feeding either on *C. reinhardtii* or *Desmodesmus* sp.** For the full model testing the effect of temperature on IS values with interaction of algae species in the form of *lm(IS ~ Temperature * Algae species)*, F_90_ = 9.97, SE = ± 0.009 , p = 0.00215, and R^2^ = 0.2717. Highlighted estimates of p-values represent significance levels lower than 0.05.

| **Response** | **Parameter** | **df** | **MS** |  | **F** | **p-value** |
| --- | --- | --- | --- | --- | --- | --- |
| *Interaction Strength* | Temperature | 1 | 0.000084 |  | 1.10 | 0.29699 |
|  | Algal species | 1 | 0.000934 |  | 12.24 | **< 0.001** |
|  | Temperature: Algal species | 1 | 0.001544 |  | 20.24 | **< 0.001** |
|  | Residuals | 90 | 0.0000763 |  |  |  |

**Table S5.** **Effect of temperature on Interaction Strength (IS) values of *D. pulex* feeding either on *C. reinhardtii* or *Desmodesmus* sp**. Model summary from the results presented in Fig. 5. Values presented here come from linear models testing the effect of temperature on IS values for each algae species in the form of: *model =* *lm(IS ~ Temperature)*. To estimate the slopes we used *emtrends(model,"Algae Species","Temperature")* and for the intercepts *emmeans(model,"Algae species",by="Temperature", at = list(Temperature = 0)* . Highlighted estimates of p-values represent significance levels lower than 0.05.

| **Algal species** | **Parameter** | **Estimate** | **SE** | **df** |  | **lower ci** | **Higher ci** | **p-value** |
| --- | --- | --- | --- | --- | --- | --- | --- | --- |
| *C. reinhardtii* | Intercept | 0.02150 | 0.00474 | 90 |  | 0.0121 | 0.03091 | **0.03** |
|  | Slope | -0.000692 | 0.000173 | 90 |  | -1.04e-03 | -0.000347 | **<0.001** |
| *Desmodesmus sp.* | Intercept | -0.00118 | 0.00472 | 90 |  | -0.0106 | 0.00821 | **0.008** |
|  | Slope | 0.000404 | 0.000171 | 90 |  | 6.43e-05 | 0.000744 | **0.02** |

**SUPPLEMENTARY METHODS**

**S. 1 – Lineage isolation, culturing and timings of experiments**

We collected all organisms (consumer and resources) from open-air freshwater mesocosms located at the River Lab, East Stoke, Dorset, UK in 2019. Mesocosms consisted of plastic tanks of approximately 1m^3^ volume and 50 cm depth that mimicked small shallow freshwater systems fluctuating naturally with air temperature (unheated ponds - Yvon-Durocher *et al.* 2011). We collected phytoplankton samples in January 2019 by serial filtration of approximately 212 L of water from eight mesocosms. We targeted the 2-30 µm fraction for small phytoplankton species. Over the next three months, we pre-isolated algal species by transferring single cells into COMBO medium (Kilham *et al.* 1998) using micro-capillary pipetting under an inverse microscope (Nikon Eclipse Ts2R). We gradually adjusted the maintenance conditions of pre-isolated populations from the field conditions, 6 ⁰C and 8L:16D photoperiod, to culture conditions at 18 ⁰C and 12L:12D over a period of five weeks in laboratory incubators (Panasonic MLR-352-PE) and with a light PAR intensity of 67 – 70 µmol m^-2^ s^-1^. Next, we streaked isolates on agar plates to reduce bacterial contamination and enhance isolation.

In April 2019, we isolated targeted algal species from our “pre-isolated” cultures using a sorting flow cytometer FACSCanto II at the CNRS Arago Laboratory, in Banyuls-sur-Mer, France. This technique allowed us to obtain pure clonal lineage cultures by sorting single cells from our highly dense and fairly clean samples of our pre-isolates. The flow cytometer allowed us to select and transfer single cells in each well of a 96-well plate containing 200 µl volume of medium. Clonal linages were maintained in the same incubator conditions previously described. A month later (May 2019) viable cultures— those where algal densities were visually high— from the clonal lineages in 96-well plates were transferred into 2 ml volume of medium and since then maintained in semi-continuous batch cultures until the start of the experiments. Maintenance consisted of transferring 10% of the old sample (20 µl) into 2 ml of fresh medium every two weeks.

In Autumn 2019 we used 18S rDNA sequencing to identify unique clonal lineages of our species. Large volumes of dense samples of clonal lineages were freeze-dried and sent to the Integrated Microbiome Resource lab at Dalhousie University (Halifax, Canada) for extraction, library preparation, and sequencing according to the procedures described in Comeau, Douglas and Langille, (2017). Following PCR-amplification of amplicon fragments, PacBio Sequel 2 long-read sequencing using full-length 18S fusion primers (forward: NSF4/18 = CTGGTTGATYCTGCCAGT; reverse: EukR = TGATCCTTCTGCAGGTTCACCTAC) identified algal clones to species and discriminated among unique clones. We identified one unique clone for each of the two species, *Chlamydomonas reinhardtii* and *Desmodesmus* sp. These clones were subsequently selected for the experiment and kept in semi-continuous batch cultures until the start of the experiments.

In March 2020, incubator temperature of clonal stock cultures was raised to 20⁰C. Finally, tests of acute thermal range for algae respiration and net photosynthesis took place in November 2020. Therefore, resource batch cultures were kept under exponential, nutrient-replete conditions in COMBO freshwater medium at 20⁰C for approximately eight months before physiological data collection.

Clonal cultures of the consumer, *Daphnia pulex*, originated from the same experimental mesocosms. We collected zooplankton samples from Dorset mesocosms by serial filtration (> 500 µm) in October 2019. Identification of daphnids to species level was by means of morphological features (Bledzki & Rybak 2016) using a digital stereo microscope (Leica S9i) and confirmed by a freshwater taxonomist (pers. comm. Dr. Antonio Guillén Oterino, April 9, 2020). From these samples, we isolated five gravid females, that constituted the five clonal lineages, of *Daphnia pulex* by placing them in Erlenmeyer flasks in modified nutrient-free COMBO medium (to prevent bacterial proliferation) in incubators at 18⁰C. Identical culturing conditions to the algae stock cultures ensured that consumers and resources experienced identical light and temperature conditions prior to experiments.

*Daphnia* were fed daily (M-F) with a homogenous mixed algal suspension consisting of *C. reinhardtii*, *Desmodesmus* sp. and *Scenedesmus obliquus* isolated from same Dorset mesocosms and maintained individually in semi-continuous batch cultures in COMBO medium in identical culturing conditions. The daily feeding regime consisted of adding a highly dense suspension of mixed algae comprising about a 10 % of total culture volume. Every week we filtered approximately 2/3 of each clonal stock culture volume in order to remove waste materials, possible kiromones, as well as any ephippia if present, which may induce diapause of cladocera. We then added an equivalent volume of fresh modified nutrient-free medium and the corresponding daily algal suspension. To reduce algae sedimentation, *D. pulex* clonal stock cultures were maintained on an orbital shaker (Heidolph Unimax 2010, orbit 20 mm, at 90-100 rpm) inside the incubators. As with the algal cultures, from March 2020 onward, we raised the culture temperature to 20⁰C. Tests of acute thermal response for *Daphnia* respiration and ingestion rates took place in June-July 2020, so consumers were kept under culturing conditions at 20⁰C over three-four months before experiments took place.

**S.2 - Starvation of algal cells and daphnids**

Nutrient starvation of algal cells started 48 hours before the experiments, with field-collected stock clonal cultures at the mid-logarithmic phase. Algal starvation was obtained by concentrating 50 ml aliquots of each algal species using an orbital centrifuge at 3,000 rpm over 15 minutes (Shirwastav *et al.* 2014; Boonchai *et al.* 2015). Then, supernatant was discarded (nutrient-replete COMBO medium) and replaced by the same volume with modified nutrient-free COMBO medium (i.e., concentrations of phosphorus and nitrogen were zero). Cells were then re-suspended in the new nutrient-fee medium and the process was repeated two more times. Finally, cultures were brought back to culturing conditions (12:12 D: L photoperiod at 20°C) in the depleted-nutrient media for 48-hours until further experiments. The main goal of this procedure was to deplete nitrogen and phosphorus pools within the cells to get as close as possible to basal metabolic conditions over the course of the experiments. Mean densities of nutrient-depleted algal cells employed for resources’ respiration and net photosynthesis experiments were set to about 60,000 cells/ml for *C. reinhardtii* and 215,000 cells/ml for *Desmodesmus sp*., based on previous pilot tests. For *Daphnia* samples we starved 5 adult daphnids— one from each clonal batch culture— for each replicate in culture tubes containing only media at culturing conditions for c. 24 hours. Thus, each replicate contained a total of 5 adult daphnids comprised of 1 daphnid from each clonal lineage. This starvation time is considered the standard for hind-gut clearance on *D. pulex* (Schlechtriem *et al.* 2006).

**S.3 – Algae preservation by freezing and flow cytometry methods**

We cryopreserved algal samples for later analysis by flow cytometry whenever algae density was a response variable [similar to (Bestion *et al.* 2021)]. Instances included algal samples collected at the end of algal respiration and net photosynthesis rates, as well as initial and final algal densities sampled from *D. pulex* ingestion rates. This procedure enabled us to accurately batch process a large number of samples collected across days and experiments using flow cytometry. For each replicate, a 150 μL sample of well-mixed algae suspension was preserved using 15 μL of 1% sorbitol solution individually in wells of a 96-well plate and incubated for about 1 hour in the dark before being frozen at - 80 °C. Sorbitol protects algal cell walls during the process of deep freezing, preventing the cells from lysing. For batch processing, sample plates were later defrosted at ambient room temperature (~ 20 – 25 ⁰C) and analysed by flow cytometry (BD FACSCanto II high throughput sampler) to estimate cell densities. Parameters of flow cytometric lasers were optimized as follows: FSC: 136, SSC: 258, FITC: 484, PE: 432, PerCP-Cy5: 297, PE-Cy7: 570, APC: 440, APC-H7: 472, V450: 373 and V500: 484. Sample plates were run on the flow cytometer at a 0.5 μL /s flow rate, with two times 100 μL mixing and a cleaning volume of 200 μL between each individual sample to avoid carry-over contamination between consecutive samples. The flow cytometer processed a sample volume of 10 μL from each replicate sample well. Then, we extracted algal density counts from flow cytometry files using the Bioconductor ‘flowCore’ (Ellis *et al.* 2020) and ’tidyverse’ (Wickham *et al.* 2019) R-packages. Extracted data provided information on cell counts and cytometric laser properties such as red fluorescence (PerCP.Cy5), a proxy of chlorophyll-a content, and forward scatter (FSC) a proxy of cell size. To remove counts of potential bacteria or debris present in our samples that would otherwise cause overestimates of algal density, we filtered the data by removing any cell count where log10(PerCP.Cy5) < 2 and log10(FSC) < 3.4. These threshold values were below minimum observed values for live cells of tested species. For algal density counts, two technical replicates were routinely collected for each sample replicate, so final density counts were obtained as the mean of these two measurements. Finally, as sample replicates were diluted by 10% with the sorbitol solution for cryopreservation, cytometer densities obtained were corrected by a factor dilution of 1.1 in order to calculate the real cell densities.

**S.4 – Data manipulation and R packages employed**

We used R statistical software (4.0.3) (R Core Team 2021) for data manipulation, analysis and plotting. The ‘tidyverse’ package was the primary package used for data manipulation and ‘ggplot2’ (Wickham 2016) for creating the graphs.

As part of *Daphnia* ingestion rates’ experiments, we verified that initial algal densities did not differ statistically between controls and consumer treatments within temporal blocks using Post-Hoc pairwise tests (i.e., for all post-hoc tests p > 0.05). For this we used ‘emmeans’ and ‘pairs’ functions from the ‘emmeans’ package (Lenth 2020). No significance difference between these treatments at the beginning of the experiment implied that any difference at the end of the experiment were only caused by the presence of the consumers. As part of the data processing, we removed two extreme opposite outliers in the ingestion rate values for *D. pulex* feeding on *C. reinhardtii* at 36 ⁰C, one from each temporal block: replicates D1 and D5 (n= 4) and one replicate for ingestion on *Desmodesmus* sp. at 36 ⁰C. We similarly removed two replicates in the interaction strength data, one at 22⁰C for *Desmodesmus* sp. and one at 36⁰C for *C. reinhardtii*. To identify outliers, we analysed distributions, means and variances of the datasets. Replicates that were inconsistent with dataset variances and at least three standard deviations away from the mean (minimum and maximum thresholds (T_min_, T_max_ = mean *± 3* *⁎ SD)* for each algal species were considered outliers. A possible explanation for these outliers could be due to a lack of precision in homogenising the samples before sampling, so we considered that they were likely related to methodological errors.

Thermal performance curves were fit using ‘rTPC’, ‘nls.multstart’ (Padfield *et al.* 2021) and ‘minpack.lm’ (Elzhov *et al.* 2016) packages. Bootstrapping using the case resampling method employing ‘rTPC’, ‘nls.multstart’ and ‘car‘ (Fox & Weisberg 2019) packages yielded confidence intervals of TPC parameters.

We fitted values of interaction strength to general linear models (GLM) by conducting a full linear regression model with interaction between temperature and algal species (*Interaction Strength ~ Temperature * Algal species*), performed a two-way analysis of variance (ANOVA), as well as obtained slopes and intercepts for each algal species and summary statistics using ‘*emtrends*’ function from ‘emmeans*’* and ‘stats*’* (R Core Team 2021) R-packages.

**S.5 – Conversion of consumer gain and loss rates into energetic equivalents**

**Conversion of Daphnia respiration rates to energetic equivalents**

Per capita consumer respiration rate, $R_{c}$, is calculated as the difference in slopes of oxygen concentration with time in consumer and control replicates:

$R_{C}= \frac{-1 \left( m_{t}-\bar{m_{c}} \right)}{d}$ (eq. S1)

where $m_{t}$ is the slope of the linear regression between oxygen saturation and time for replicates containing *Daphnia* adults, $\bar{m_{c}}$ is the mean slope of controls for each temporal block, and *d* is the number of adult daphnids alive at the beginning of the experiment in *Daphnia* treatment replicates. The value of *d* was used to control for mortality. It was generally 5, but in exceptional cases consumer numbers dropped during the acclimation ramp-up.

Therefore, units of per capita respiration rates are:

$\left[ R_{C} \right]=\frac{\frac{mg O_{2}}{L \times min}}{Daphnia ind.}=\frac{mgO_{2}}{L*min*D ind}$ (eq. S2)

We next transformed per capita respiration rate, $R_{C}$, to an energy equivalent, $R_{C}^{E}$, in order to make respiration rates comparable to ingestion rates, with units:

$\left[ R_{C}^{E} \right]=\frac{J}{L*h*D ind}$

Hence, we used the following transformation:

$\left[ R_{C}^{E} \right]=\frac{J}{mgO_{2}}*\frac{min}{h}*[R_{C}]$

We assumed that 1 mg of O_2_ equals 14.06 Joules of energy (Peters 1983; Rall *et al.* 2010) and converted minutes to hours by multiplying $R_{C}$ by 60:

Thus, we have:

$R_{C}^{E}=14.06 \frac{Joules}{mg O_{2}}\times60 \frac{min}{h} {\times R}_{C}= 843.6\frac{J*min}{mgO_{2}*h} \times R_{C}$ (eq. S3)

**Conversion of *Daphnia* ingestion rates to energetic equivalents**

Per capita raw ingestion rate, $rI$, is measured as the difference in resource density at the beginning and the end of the experiment in the presence and absence of consumers (consumer and control replicates respectively) and corrected for consumer numbers. Therefore:

$rI= \frac{\left( D_{TO}-D_{T15O} \right)+\left( \bar{C_{150}- C_{0}} \right)}{y t}$ (eq. S4)

where D_T0_ = algal densities in the *Daphnia* replicates at the beginning of the experiment (T0), D_T150_ = algal densities in the *Daphnia* replicates at the end of the experiment (T150), $\bar{C_{0}}$ is the mean value of algal densities at the beginning of the experiment (T0) in controls within each of 2 temporal blocks, $\bar{C_{150}}$ is the mean value of algal densities at the end of the experiment (T150) in controls within each of 2 temporal blocks, $y$ = the number of daphnids introduced into each *Daphnia* replicate, and $t$ is the length of the experiment, 2.5 hours.

Hence, raw ingestion rate has the following units:

$\left[ rI \right]=\frac{\frac{cells}{mL}}{h*Daphnia ind.}=\frac{cells}{mL*h*D ind}$ (eq. S5)

We next transformed per capita raw ingestion rate, $rI$, into energetic equivalents of per capita raw ingestion rates, ${rI}^{E}$, in order to make ingestion rates (i.e., energy gain) comparable to respiration rates (i.e., energy loss). Units of per capita raw ingestion rates in energy equivalents, ${rI}^{E}$, are:

$\left[ {rI}^{E} \right]=\frac{J}{L*h*D ind}$ (eq. S6)

We used the following transformation

$${[ rI}^{E}]=\left[ rI \right]\times\left[ Biovolume \right]\times\left[ Biomass cf \right]\times[Calorific value] \times\frac{mL}{L}$$

Which means:

$[{rI}^{E}]=\frac{cells}{mL*h*D ind}\times\frac{{\mu m}^{3}}{cells}\times\frac{g}{{\mu m}^{3}}\times\frac{J}{g}\times\frac{mL}{L}=\frac{J}{L*h*D ind}$ (eq. S7)

The conversion from mL to L, simply requires multiplication by 1000. The specific conversion values for biovolume, biomass and calorific value depend upon which resource species upon which the *Daphnia* feeds. When *D. pulex* feeds on *Chlamydomonas reinhardtii*, we calculated the biovolume of our samples as 343 µm^3^/cell using manual measurements following the methods of Hillebrand *et al.*, (1999). The biomass conversion factor used was 0.28 10^-12^ g/µm^3^ (Montagnes *et al.* 2001; Finkel *et al.* 2016). And finally, the calorific value for this species (i.e., the amount of energy provided in Joules per gram) is equal to 22.397 x 10^3^ Joules/g (Feinberg 1984). Hence, multiplying the specific values of biovolume, the biomass conversion factor, the calorific value and the volume correction factor for *C. reinhardtii* yields a conversion factor of 2.15 x 10^-3^ to get energy equivalents of ingestion rates:

${rI}_{C. reindhartii}^{E}=343\frac{{\mu m}^{3}}{cells}\times0.28*{10}^{-12}\frac{g}{{\mu m}^{3}}\times22.397*{10}^{3}\frac{J}{g}\times{10}^{3}\frac{mL}{L}\times rI= \boldsymbol{2.15}*{10}^{-3} \frac{{\mu m}^{3}}{cells}\times\frac{g}{{\mu m}^{3}}\times\frac{J}{g}\times\frac{mL}{L}\times rI$ (eq. S8)

When *D. pulex* feeds on *Desmodesmus* sp., the calculated biovolume of our samples [following Hillebrand *et al.*, (1999b)] was 310 µm^3^/cell. The biomass conversion factor used was 0.139 10^-12^ g/µm^3^ (Rocha & Duncan 1985) , based on *Scenedesmus quadricauda.* This species is the most similar species [i.e., in terms of morphology and biovolume (≃ 270 µm^3^/cell)] to our *Desmodesmus* species for which a biomass conversion factor was available in the literature. The calorific value used for this species was 23 x 10^3^ Joules/g (Tibbetts *et al.* 2016). The aggregated conversion factor for *Desmodesmus* sp. to get energy equivalents of ingestion rates is thus 0.99 x 10^-3^:

${rI}_{Desmodesmus sp.}^{E}=\boldsymbol{0.99}*{10}^{-3} \frac{{\mu m}^{3}}{cells}\times\frac{g}{{\mu m}^{3}}\times\frac{J}{g}\times\frac{mL}{L}\times rI$ (eq. S9)

Finally, as not all the algal cellular components ingested by the consumer will be assimilated, we calculated the final per capita ingestion rates, $I^{E}$, by multiplying per capita raw ingestion rates in energy units, ${rI}^{E}$, by a unitless assimilation efficiency $(W)$:

$I^{E} \left[ \frac{\mathrm{Joules}}{Daphnia\times L \times h} \right]={rI}^{E}*W (\%)$ (eq. S10)

We assumed that assimilation efficiencies remained constant across temperatures with $W=$14% when *D. pulex* fed upon *C. reinhardtii* (Wetzel 2001) and $W=$10% when it instead fed upon *Desmodesmus* sp. Information on the assimilation efficiency of *D. pulex* feeding on *Desmodesmus* sp. in the literature is scarce, but assuming that this species has a different cell wall chemical composition compared to *C. reinhardtii*— larger quantities of carbon fixed in the cell wall are in form of cellulose and pectin— this suggests that *Desmodesmus* is likely more difficult to assimilate for the consumer compare to *C. reinhardtii*. For this reason*,* we assume that assimilation efficiency must be lower for this species. Additionally, It has been reported that *D. pulex* feeding on *Scenedesmus acuminatus* shows an assimilation efficiency of near 7% (Infante 1973). This species has more intricate cell structures than our species of *Desmodesmus*. For this reason, we considered that assimilation efficiency for *D. pulex* feeding on *Desmodesmus* sp. should be somewhere between 7 and 14%. Therefore, we conservatively used 10% as a constant value of assimilation efficiency for *Desmodesmus* sp.

**Supplementary References**

Bestion, E., Haegeman, B., Alvarez Codesal, S., Garreau, A., Huet, M., Barton, S., *et al.* (2021). Phytoplankton biodiversity is more important for ecosystem functioning in highly variable thermal environments. *Proc. Natl. Acad. Sci. U. S. A.*, 118.

Bledzki, L.A. & Rybak, J.I. (2016). *Freshwater Crustacean Zooplankton of Europe*. Springer International Publishing, Cham.

Boonchai, R., Kaewsuk, J. & Seo, G. (2015). Effect of nutrient starvation on nutrient uptake and extracellular polymeric substance for microalgae cultivation and separation. *Desalin. Water Treat.*, 55, 360–367.

Comeau, A.M., Douglas, G.M. & Langille, M.G.I. (2017). Microbiome Helper: a Custom and Streamlined Workflow for Microbiome Research. *mSystems*, 2.

Ellis, B., Haaland, P., Hahne, F., Nolwenn {Le Meur}, Gopalakrishnan, N., Spidlen, J., *et al.* (2020). flowCore: flowCore: Basic structures for flow cytometry data. R package version 2.2.0.

Elzhov, T. V., Mullen, K.M., Spiess, A.-N. & Ben Bolker. (2016). minpack.lm: R Interface to the Levenberg-Marquardt Nonlinear Least-Squares Algorithm Found in MINPACK, Plus Support for Bounds. R package version 1.2-1.

Feinberg, D.A. (1984). *Fuel Options from Microalgae with Representative Chemical Composition*. Goiden, Colorado.

Finkel, Z. V., Follows, M.J. & Irwin, A.J. (2016). Size-scaling of macromolecules and chemical energy content in the eukaryotic microalgae. *J. Plankton Res.*, 38, 1151–1162.

Fox, J. & Weisberg, S. (2019). *An R Companion to Applied Regression*. Third. Sage, Thousand Oaks CA.

Hillebrand, H., Dürselen, C.D., Kirschtel, D., Pollingher, U. & Zohary, T. (1999). Biovolume calculation for pelagic and benthic microalgae. *J. Phycol.*, 35, 403–424.

Infante, A. (1973). Investigations on the ’incorporation of different algae by some zooplankton species - Ùntersuchungen über die Ausnutzbarkeit verschiedéner Algen durch das gooplankton -. *Arch. FUR Hydrobiol.*, 42, 340–405.

Kilham, S.S., Kreeger, D.A., Lynn, S.G., Goulden, C.E. & Herrera, L. (1998). COMBO: A defined freshwater culture medium for algae and zooplankton. *Hydrobiologia*, 377, 147–159.

Lenth, R. V. (2020). emmeans: Estimated Marginal Means, aka Least-Squares Means. R package version 1.5.3.

Montagnes, D.J.S., Kimmance, S., Tsounis, G. & Gumbs, J.C. (2001). Combined effect of temperature and food concentration on the grazing rate of the rotifer Brachionus plicatilis. *Mar. Biol.*, 139, 975–979.

Padfield, D., O’Sullivan, H. & Pawar, S. (2021). rTPC and nls.multstart: A new pipeline to fit thermal performance curves in r. *Methods Ecol. Evol.*, 12, 1138–1143.

Peters, R.H. (1983). *Ecological implications of body size*. Cambridge University Press.

R Core Team. (2021). R: A Language and Environment for Statistical Computing. R Foundation for Statistical Computing.

Rall, B.C., Vucic-pestic, O., Ehnes, R.B., Emmerson, M. & Brose, U. (2010). Temperature , predator – prey interaction strength and population stability. *Glob. Chang. Biol.*, 16, 2145–2157.

Rocha, O. & Duncan, A. (1985). The relationship between cell carbon and cell volume in freshwater algal species used in zooplanktonic studies. *J. Plankton Res.*, 7, 279–294.

Schlechtriem, C., Arts, M.T. & Zellmer, I.D. (2006). Effect of temperature on the fatty acid composition and temporal trajectories of fatty acids in fasting Daphnia pulex (Crustacea, Cladocera). *Lipids*, 41, 397–400.

Shirwastav, A., Gupta, S.K., Ansari, F.A., Rawat, I. & Bux, F. (2014). Adaptability of growth and nutrient uptake potential of Chlorella sorokiniana with variable nutrient loading. *Bioresour. Technol.*, 174, 60–66.

Tibbetts, S.M., Melanson, R.J., Kyoung, C.P., Banskota, A.H., Stefanova, R. & McGinn, P.J. (2016). Nutritional Evaluation of Whole and Lipid-Extracted Biomass of the Microalga Scenedesmus sp. AMDD Isolated in Saskatchewan, Canada for Animal Feeds: Proximate, Amino Acid, Fatty Acid, Carotenoid and Elemental Composition. *Curr. Biotechnol.*, 4, 530–546.

Wetzel, R.G. (2001). *Limnology: Lake and River Ecosystems*. *J. Phycol.* Academic press.

Wickham, H. (2016). *ggplot2: Elegant Graphics for Data Analysis*. Springer-Verlag New York.

Wickham, H., Averick, M., Bryan, J., Chang, W., McGowan, L., François, R., *et al.* (2019). Welcome to the Tidyverse. *J. Open Source Softw.*, 4, 1686.

Yvon-Durocher, G., Montoya, J.M., Trimmer, M. & Woodward, G. (2011). Warming alters the size spectrum and shifts the distribution of biomass in freshwater ecosystems. *Glob. Chang. Biol.*, 17, 1681–1694.
